# Discovering comorbid diseases using an inter-disease interactivity network based on biobank-scale PheWAS data

**DOI:** 10.1093/bioinformatics/btac822

**Published:** 2022-12-26

**Authors:** Yonghyun Nam, Sang-Hyuk Jung, Jae-Seung Yun, Vivek Sriram, Pankhuri Singhal, Marta Byrska-Bishop, Anurag Verma, Hyunjung Shin, Woong-Yang Park, Hong-Hee Won, Dokyoon Kim

**Affiliations:** Department of Biostatistics, Epidemiology & Informatics, Perelman School of Medicine, University of Pennsylvania, Philadelphia, PA 19104, USA; Department of Biostatistics, Epidemiology & Informatics, Perelman School of Medicine, University of Pennsylvania, Philadelphia, PA 19104, USA; Department of Digital Health, SAIHST, Sungkyunkwan University, Samsung Medical Center, Seoul 06355, Republic of Korea; Department of Biostatistics, Epidemiology & Informatics, Perelman School of Medicine, University of Pennsylvania, Philadelphia, PA 19104, USA; Division of Endocrinology and Metabolism, Department of Internal Medicine, St. Vincent’s Hospital, College of Medicine, The Catholic University of Korea, Seoul 06591, Republic of Korea; Department of Biostatistics, Epidemiology & Informatics, Perelman School of Medicine, University of Pennsylvania, Philadelphia, PA 19104, USA; Department of Genetics, Perelman School of Medicine, University of Pennsylvania, Philadelphia, PA 19104, USA; New York Genome Center, New York, NY 10013, USA; Department of Medicine, Perelman School of Medicine, University of Pennsylvania, Philadelphia, PA 19104, USA; Department of Artificial Intelligence, Ajou University, Suwon 16499, Republic of Korea; Samsung Genome Institute, Samsung Medical Center, Sungkyunkwan University School of Medicine, Seoul 06351, Republic of Korea; Department of Digital Health, SAIHST, Sungkyunkwan University, Samsung Medical Center, Seoul 06355, Republic of Korea; Samsung Genome Institute, Samsung Medical Center, Sungkyunkwan University School of Medicine, Seoul 06351, Republic of Korea; Department of Biostatistics, Epidemiology & Informatics, Perelman School of Medicine, University of Pennsylvania, Philadelphia, PA 19104, USA; Institute for Biomedical Informatics, University of Pennsylvania, Philadelphia, PA 19104, USA

## Abstract

**Motivation:**

Understanding comorbidity is essential for disease prevention, treatment and prognosis. In particular, insight into which pairs of diseases are likely or unlikely to co-occur may help elucidate the potential relationships between complex diseases. Here, we introduce the use of an inter-disease interactivity network to discover/prioritize comorbidities. Specifically, we determine disease associations by accounting for the direction of effects of genetic components shared between diseases, and categorize those associations as synergistic or antagonistic. We further develop a comorbidity scoring algorithm to predict whether diseases are more or less likely to co-occur in the presence of a given index disease. This algorithm can handle networks that incorporate relationships with opposite signs.

**Results:**

We finally investigate inter-disease associations among 427 phenotypes in UK Biobank PheWAS data and predict the priority of comorbid diseases. The predicted comorbidities were verified using the UK Biobank inpatient electronic health records. Our findings demonstrate that considering the interaction of phenotype associations might be helpful in better predicting comorbidity.

**Availability and implementation:**

The source code and data of this study are available at https://github.com/dokyoonkimlab/DiseaseInteractiveNetwork.

**Supplementary information:**

Supplementary data are available at *Bioinformatics* online.

## 1 Introduction

Comorbidity describes when a given patient has one or more additional medical conditions co-occurring alongside a primary disease (Lee *et al.*, 2008; Valderas *et al.*, 2009). Predicting comorbidity is important for planning the clinical care of individual patients and for investigating clinical epidemiology; in particular, mortality risk is often increased when patients with underlying diseases are also diagnosed with adverse medical outcomes or complications (Cho *et al.*, 2021; Jørgensen *et al.*, 2012; Nashiry *et al.*, 2021). Comorbidity studies involving multiple phenotypes at the population level can be divided into two categories according to the approach employed: (i) statistical analysis based on disease prevalence, or (ii) network analysis based on sharing of components among diseases. Notable studies using the statistical approach include Buddeke *et al.* (2019), a cohort study based on clinical records that presented complications with cardiovascular diseases (Buddeke *et al.*, 2019; Klimek *et al.* (2015), which used nationwide claim data to identify diabetes-related comorbidities (Klimek *et al.*, 2015); and Richardson *et al.* (2020), which revealed conditions comorbid with SARS-CoV-2 among 5700 hospitalized patients (Richardson *et al.*, 2020). Similarly, studies using network-based approaches and disease–disease associations include: Hidalgo *et al.* (2009), which built comorbidity maps for 657 diseases from the clinical records of over 30 million patients, with connectivity defined by prevalence-based comorbidity measures (Hidalgo *et al.*, 2009; Rubio-Perez *et al.* (2017), which explored potential comorbidity relationships by constructing a disease–disease network (DDN) based on shared disease-associated genes and their functions (Rubio-Perez *et al.*, 2017; Verma *et al.* (2019), which similarly identified disease comorbidities on the basis of shared genetic components by constructing a DDN based on a phenome-wide association study (PheWAS) that used electronic health record (EHR)-linked biobank data (Verma *et al.*, 2019; Dong *et al.*, 2021), which discovered disease multimorbities using hospital inpatient data in the UK Biobank and provided biological explanations by constructing disease network with a set of genome-wide association studies (GWASs) data (Dong *et al.*, 2021).

Separately, several studies have attempted to categorize comorbidity as being direct or inverse (Ibáñez *et al.*, 2014; Sánchez-Valle *et al.*, 2020; Tabarés-Seisdedos and Valderas, 2013; Tarantino *et al.*, 2018); i.e. given an underlying disease, conditions that frequently co-occur at the population level are considered directly comorbid, while those with relatively few co-occurrences have inversely comorbid. Catalá-López *et al.* discovered that Alzheimer’s disease can decrease the co-occurrence of cancers from meta-analysis with literatures (Catalá-López *et al.*, 2014). Roitmann *et al.* revealed inverse comorbidity relationships from clustering analysis with health records in Demark (Roitmann *et al.*, 2014).

DDNs represent a map of topologies between phenotypes having shared biological components that may indicate phenotypic similarity and comorbidity. Several DDNs were constructed to provide insight into the human disease interactome by using genetic components shared with diseases, such as genes, proteins, or pathways (Barabási *et al.*, 2011; Goh *et al.*, 2007; Liu *et al.*, 2014; Zhou *et al.*, 2014). Other studies have used summary statistics obtained from GWASs to observe disease–disease associations by leveraging disease-associated single nucleotide polymorphisms (SNPs) (Darabos *et al.*, 2015; Dong *et al.*, 2021; Nam *et al.*, 2022; Verma *et al.*, 2019). However, these previous association-based DDN studies have not considered whether the associations or interactions that depend on shared components are agonistic or antagonistic. As shared genetic components can have different effects on different disease mechanisms, incorporating this directional information can improve the ability to predict comorbidity. For example, cardiovascular diseases have a synergistic association with low-density lipoprotein cholesterol (LDL-C) and an antagonistic association with high-density lipoprotein cholesterol (HDL-C): high LDL-C may increase the risk of cardiovascular disease, while high HDL-C may reduce it (Di Angelantonio *et al.*, 2009; Sharrett *et al.*, 2001). Insight into which disease pairs are likely or unlikely to co-occur may help in understanding the potential relationships of complex diseases. Moreover, as our knowledge of biological mechanisms advances, further genetic components of risk factors or protective factors related to disease mechanisms are revealed (von Mutius and Smits, 2020; Zhu *et al.*, 2018). Notably, Bulik-Sullivan *et al.* (2015) successfully identified negative genetic correlations that agreed with epidemiological associations (negatively correlated traits co-occur less than expected by chance), and also identified phenotypic correlations between complex traits and diseases via LD Hub and PhenoSpD as a follow-up study (Bulik-Sullivan *et al.*, 2015; Zheng *et al.*, 2017, 2018). They utilized linkage disequilibrium score (LDSC) regression to estimate the strength of genetic correlations between diseases by comparing association test statistics between two diseases according to LD score trait in a polygenic model. Therefore, LDSC-based methods effectively calculate genetic correlations mainly for polygenic traits, using all variants regardless of their statistical significance in GWAS (Bulik-Sullivan *et al.*, 2015). Because quantifying genetic correlations using LDSC regression focused on the entire spectrum of variants in GWASs, it is hard to directly apply their method to estimate disease interactions based on knowledge-based shared genetic components (e.g. disease–gene associations cataloged in the Online Mendelian Inheritance in Man database) or data-driven shared components with statistical thresholds (e.g. based on significant variants in genome-wide data).

The association-based DDNs that consider only the total amount of shared components are not easy to determine whether the relationships between diseases are agonistic or antagonistic. In contrast, the correlation-based DDNs computed by LDSC regression can determine disease interactions but it may include more false-positive disease associations in the resulting networks than those considering only significant SNPs. This motivates us to explore interactions between diseases considering the direction of effect of shared genetic components that reach genome-wide significance in PheWAS summary statistics and to study how inter-disease interactivity is related at the epidemic and genomic levels.

Here, we propose a novel network-based framework to build an inter-disease interaction network that can take into account the direction of effects for components shared between diseases. We consider phenotypes as having either synergistic or antagonistic association: synergistic if two diseases mutually increase the tendency of their co-occurrence, or antagonistic if they decrease the tendency of co-occurrence. To examine whether the interactivity of disease associations can help predict/prioritize comorbidity, we constructed a DDN that considers the direction of effect of SNPs significantly associated with diseases, obtained from PheWAS summary statistics. This inter-disease interactivity network is a signed disease–disease network (signed DDN) having both positive and negative edges in the graph (i.e. positive values for a synergistic association and negative for an antagonistic association). To translate synergistic and antagonistic association at the genomic level to direct and inverse comorbidity at an epidemic level, we also develop a novel graph-based semi-supervised learning (SSL) for comorbidity scoring, modifying the objective functions of label propagation algorithms to work for a signed DDN with a unary label by introducing a signed degree matrix and signed graph Laplacian (Gallier, 2016). The scoring algorithm prioritizes comorbid diseases in relation to an index disease of interest.

## 2 Materials and methods

**Fig. 1.**
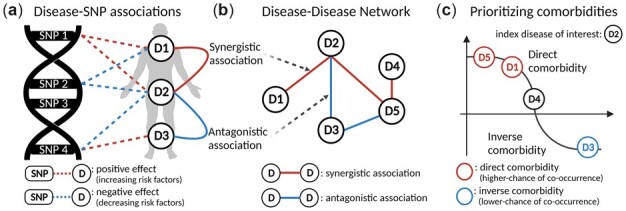
Schematic description of the proposed method. (**a**) Defining directionality of disease–SNP associations. D1 and D2 have a synergistic association because the overall effects of their shared SNPs have consistent direction, whereas D2 and D3 have an antagonistic association because the effects of their shared SNPs have opposite direction. (**b**) DDN incorporating synergistic and antagonistic associations. We constructed a signed DDN with pre-defined/pre-calculated disease associations. (**c**) Prioritizing comorbidity. Higher scores mean higher chance of disease co-occurrence; D5 has the highest chance of comorbidity with D2

The overall procedure consists of three main parts (Fig. 1): (i) constructing the signed DDN based on shared genetic components, (ii) predicting comorbidity scores using graph-based SSL and (iii) prioritizing/ranking comorbid conditions in relation to a given disease.

First, we built a signed DDN based on the sharing of significant SNPs between disease pairs, which relied on data obtained from UK Biobank PheWAS summary statistics. We determined disease associations by accounting for the overall direction of effects of genetic components shared between diseases, and categorize those associations as synergistic or antagonistic. In this network, two diseases have a synergistic association if their shared SNPs have the overall same direction of effect, or an antagonistic association if the SNP effects are in opposition. For example, Disease 1 (D1) and Disease 2 (D2) have shared components in SNP1 and SNP2, whereas D2 and D3 share SNP4 (Fig. 1a). Both SNP1 and SNP2 display the same direction of effect on D1 and on D2: SNP1 is associated with positive direction of effects (increasing risk), while SNP2 is associated with negative direction of effects (decreasing risk). Thus, the association between D1 and D2 is synergistic. In contrast, D2 and D3 have an antagonistic association because the respective effect of SNP4 is opposite (i.e. positive for D2 but negative for D3). Once the associations are determined, a signed DDN is constructed to represent the relationships between phenotype pairs as a graph with nodes and edges (Fig. 1b).

Second, we perform comorbidity score prediction: given a specific disease, we predict comorbidity of other diseases by applying graph-based SSL to the constructed network (Lee *et al.*, 2020; Nam *et al.*, 2019a,b). Since the network includes both synergistic and antagonistic associations (edge weights with positive and negative values), we posited the following hypotheses concerning comorbidity predictions: (i) two diseases have a chance of comorbidity if they are connected (i.e. have at least one shared SNP), regardless of association direction; (ii) if two diseases have a synergistic association according to overall direction of shared SNPs, they are more likely to co-occur (high chance of co-occurrence); and (iii) if two diseases have an antagonistic association, they are less likely to co-occur (low chance of co-occurrence). If these hypotheses are supported, scoring algorithms can predict the comorbidity of another medical condition given a specific underlying disease.

Finally, we prioritize the chance of disease co-occurrence based on the predicted comorbidity scores, stratifying by deciles. All diseases in a network have some chance of comorbidity with the index disease, but the degree of association can range considerably; a representative example is illustrated in Figure 1c. This ranking is then validated using disease prevalence-based co-occurrence measures estimated from EHRs (Hidalgo *et al.*, 2009).

### 2.1 Constructing the DDN from PheWAS summary data

**Fig. 2.**
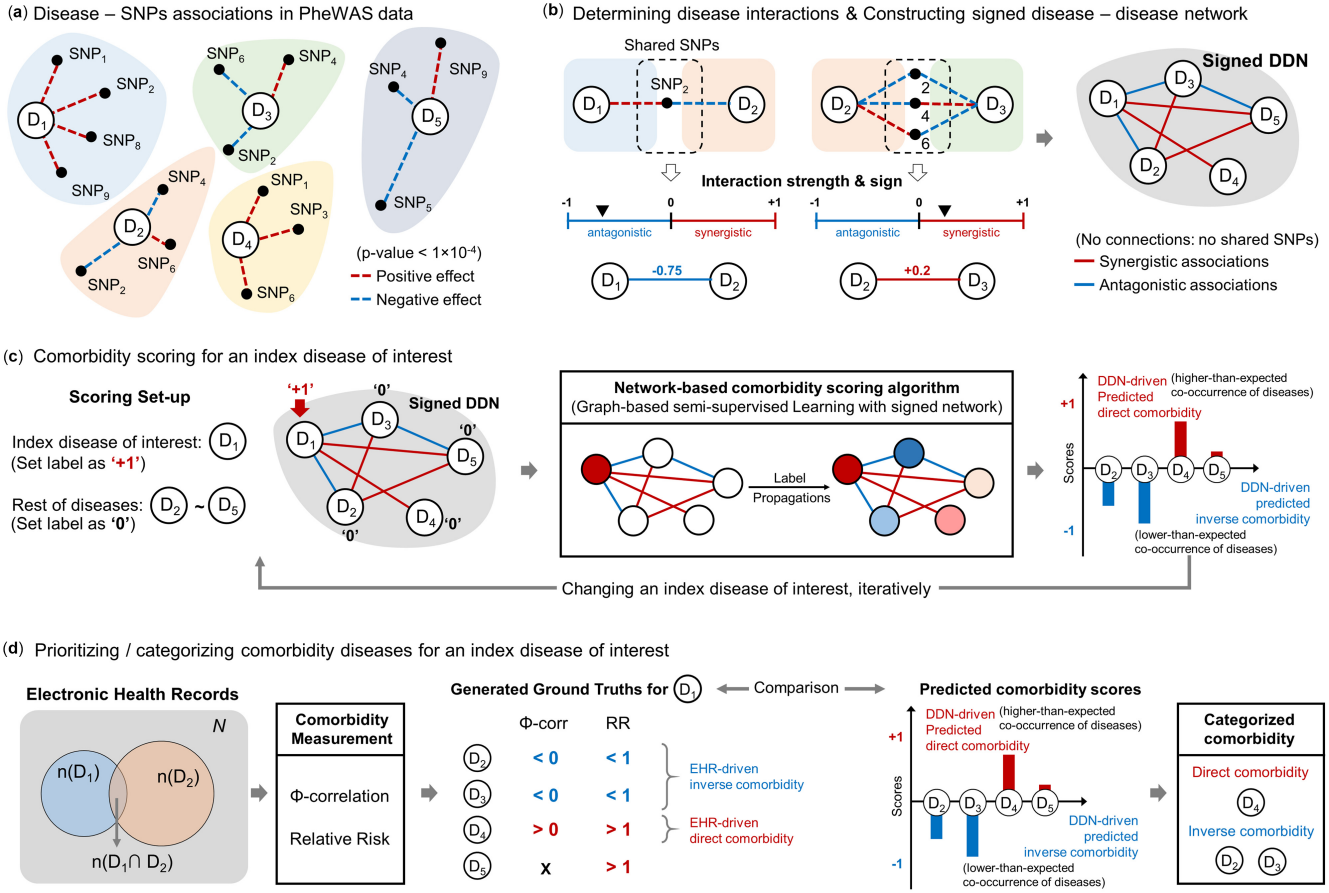
Step-by-step explanation of the proposed method. (**a**) Visualization of disease–SNPs associations in PheWAS summary data. The positive/negative direction of effect of SNPs (dashed line) depicts the direction of beta-coefficients obtained from summary statistics. (**b**) Constructing inter-disease interactive network (signed DDN) with determining synergistic and antagonistic associations. (**c**) Semantic description of proposed comorbidity scoring algorithm with graph-based SSL. (**d**) Comparison scheme with predicted DDN-driven comorbidity and EHR-driven comorbidity. Based on this explanation, we provide pseudocode in Supplementary Algorithm S1

The proposed DDN was built with a shared genetic component hypothesis, namely that two different phenotypes were linked if they shared the significant SNPs from the PheWAS summary statistics. We obtained the beta-coefficient ($\beta_{ik}$) and standard error (${\mathrm{SE}}_{ik}$) values for the association between phenotype i and SNP k when that association passed the *P*-value threshold for significance in summary statistics. Then, we selected an appropriate significance threshold (*P*-value $<\;1\times 10^{-4}$) and constructed a disease–SNP association matrix: specifically, given m phenotypes and k SNPs, we constructed the association matrix $R\in R^{m\times k}$ (Fig. 2a). For each SNP k associated with disease i, the element $r_{ik}=\beta_{ik}/SE_{ik}$ is a constant value of *z*-score consisting of the corresponding beta-coefficient divided by the standard error. The sign of $r_{ik}$ indicates the direction of effect of SNP k in relation to disease i. The DDN was then constructed from this matrix to represent the genetic associations between pairs of phenotypes by measuring proximity between disease-SNP vectors.

This DDN is an undirected, signed and weighted graph $G=\left(V,W\right)$ in which $V=\left\{v_{i}\in R^{k}\right|\;i=1,\;2,\;.,\;m\}$ denotes the set of diseases and $W=\left\{w_{ij}\;\right|\;i,j=1,\;2\ldots ,m\}$ denotes the similarity between diseases. Each disease $v_{i}=\{r_{i1},\;r_{i2},\;\ldots ,r_{ik}\}$ has k-dimensional SNP vectors, and the similarity $w_{ij}$ between any two diseases $v_{i}$ and $v_{j}$ is calculated as follows:

(1) $$w_{ij}=\frac{\sum_{k\in S}\left(r_{ik})\cdot (r_{jk}\right)}{\sqrt{\sum_{k=1}^{K}r_{ik}^{2}}\sqrt{\sum_{k=1}^{K}r_{jk}^{2}}}=\frac{\sum_{k\in S}\left(\beta_{ik}/SE_{ik}\right)\cdot \left(\beta_{jk}/SE_{jk}\right)}{\sqrt{\sum_{k=1}^{K}{{(\beta }_{ik}/SE_{ik})}^{2}}\sqrt{\sum_{k=1}^{K}{{(\beta }_{ik}/SE_{ik})}^{2}}},$$

where $r_{ik}$ and $r_{jk}$ are the respective *z*-scores associated with SNP k for diseases $v_{i}$ and $v_{j}$; K is the total number of SNPs; and S is the set of significant SNPs shared between $v_{i}$ and $v_{j}$. Since the *z*-score $r_{i}$ can take a negative value, each edge weight $w_{ij}$ can range from −1 to 1. Figure 2a illustrates the constructed disease similarity matrix (W) with both positive and negative associations. The magnitude of a weight ($\left|w_{ij}\right|$) between a disease pair describes the number of significant SNPs those diseases share. A synergistic association ($w_{ij}>0$) means that two diseases have shared SNPs and the overall effect of those SNPs has the same direction for both diseases. An antagonistic association ($w_{ij}<0$) indicates that the shared SNPs have overall opposite directions of effect for the two diseases. The similarity matrix can quantify how similar two diseases are in terms of the directions of SNP effects.

### 2.2 Network-based comorbidity scoring algorithms

Once the DDN incorporating synergistic and antagonistic associations (signed DDN) was constructed, comorbidity scoring was performed using a graph-based SSL, which is employed for scoring algorithms (Chong *et al.*, 2020; Subramanya and Talukdar, 2014). Figure 2b describes the problem setup for predicting comorbidity scores. The network-based scoring algorithm used here is a transductive learning approach (Chong *et al.*, 2020); it predicts the co-occurrences between an underlying disease and other diseases when only the underlying disease is known to occur (one positive sample), and all others have unknown occurrences (unlabeled samples). Those scores can provide prioritized comorbidity scores for the unlabeled diseases by propagating with disease associations from the signed DDN. The proposed network simultaneously contains both positive and negative edges, obtained from (1); i.e. two diseases having SNPs in common can co-occur regardless of the respective directionality of SNP effects. Nevertheless, the chance of co-occurrence for two synergistically associated diseases may be relatively higher than that of two diseases having an antagonistic association, since the former pair shares many SNPs with the same direction of effect while the latter features SNPs with opposite directions of effect.

The following describes the procedure of comorbidity scoring. Consider a signed DDN G=(V,W), where the similarity matrix W has both positive and negative edge weights. Let $y={\left(y_{1},\ldots ,y_{m}\right)}^{T}$ denotes the initial labels for the set of diseases and $f={\left(f_{1},\;\ldots ,\;f_{m}\right)}^{T}$ the set of predicted comorbidity scores. We set a unary label ($y_{l}=+1$) for an index disease of interest ($v_{l}$) and set unlabeled ($y\backslash y_{l}=\{0\})$ for remaining other diseases. Label information on the labeled disease is propagated to unlabeled diseases on graph G to obtain real-valued scores f, with two assumptions: the *smoothness condition* (predicted scores $f_{i}$ should not be too different from the $f_{j}$ values in adjacent unlabeled nodes) and the *loss condition* (predicted scores $f_{i}$ should be close to the given label of $y_{i}$). Generally, for an unsigned graph, $\tilde{G}=(V,\;\tilde{W})$, the smoothness condition is represented as

(2) $$f^{T}Lf=\sum_{i∼j}{\tilde{w}}_{ij}{\left(f_{i}-f_{j}\right)}^{2},$$

where the graph Laplacian L is defined as $L=D-\tilde{W}$, $\tilde{W}$ being the unsigned similarity matrix, $D=\mathrm{diag}(d_{i})$ the degree matrix and $d_{i}=\sum_{j}w_{ij}$. However, since the proposed network has signed edges from (1), we modified the smoothness condition of (2) to handle both positive and negative associations by incorporating a signed degree matrix:

(3) $$f^{T}\overset{-}{L}f=\;\sum_{i∼j}\left|w_{ij}\right|{\left(f_{i}-\mathrm{sign}(w_{ij})f_{j}\right)}^{2},$$

where $\bar{L}$ is the signed graph Laplacian, defined as $\bar{L}=\bar{D}-W$, in which $\bar{D}=\mathrm{diag}(\bar{d_{i}})$ is the signed degree matrix with $\overset{-}{d_{i}}=\sum_{j}\left|w_{ij}\right|$ and sign(⋅) is the signum function. The signed graph Laplacian $\bar{L}$ is positive semidefinite, and the quantity of $f^{T}\bar{L}f$ is non-negative (Gallier, 2016). The loss condition is ${\left(f-y\right)}^{T}\left(f-y\right)=\sum_{i}{\left(f_{i}-y_{i}\right)}^{2}$. The predicted output f is obtained by minimizing the following quadratic function using the loss and smoothness conditions:

(4) $$\underset{f}{\min}{\;\left(f-y\right)}^{T}(f-y)+\mu f^{T}\overset{-}{L}f.$$

The closed form solution is obtained as

(5) $$f={\left(I+\mu \overset{-}{L}\right)}^{-1}y,$$

where the hyper-parameter μ trades off loss and smoothness. The resulting predicted outcome f on unlabeled nodes has either positive or negative values. We denote the predicted comorbidity scores f as the DDN-driven comorbidity, which indicates the relative likelihood of two diseases co-occurring based on the direction of effects of genetically associated factors.

### 2.3 Prioritizing and categorizing comorbidity

We prioritize and categorize the list of co-occurring diseases as having higher or lower chance of co-occurrence. Hereafter, we denote the higher chance of comorbidity as *direct comorbidity* and the lower chance as *inverse comorbidity* (Catalá-López *et al.*, 2014; Tabarés-Seisdedos and Baudot, 2016). We categorize DDN-driven comorbidity f into direct and inverse comorbidity. For validating DDN-driven comorbidity, we used co-occurrence measures estimated from EHR (denoted as EHR-driven comorbidity). EHR-driven comorbidity is defined in terms of the disease prevalence-based ϕ-correlation and relative risk (Hidalgo *et al.*, 2009).

The Pearson correlations for binary variables (ϕ-correlation) and relative risk were used to estimate EHR-driven comorbidity (Fig. 2c). Given N patients and the list of m diagnoses in clinical records, we constructed a ($\frac{m^{2}-m}{2}$) binary contingency matrix for each disease pair to display the frequency distribution. The *ϕ*-correlation for the pair of diseases i and j is calculated by $\phi_{ij}=\frac{C_{ij}N-P_{i}P_{j}}{\sqrt{P_{i}P_{j}(N-P_{i})(N-P_{j})}}$ based on their prevalence, where $C_{ij}$ is the number of patients diagnosed with both diseases while $P_{i}$ and $P_{j}$ indicate the number of patients diagnosed with disease i and j, respectively. A positive correlation means two diseases tend to occur simultaneously and a negative correlation that they tend not to co-occur. We also captured tendency of co-occurrence among disease pairs by calculating relative risks. The relative risk for two diseases $RR_{ij}=\frac{C_{ij}N}{P_{i}P_{j}\;}$ is measured as the ratio of risk between the diseases based on the disease prevalence. ${\mathrm{RR}}_{ij}$ > 1 indicates that both diseases co-occur more frequently than expected by chance, and ${\mathrm{RR}}_{ij}$ < 1 indicates their less frequent co-occurrence than expected by chance (Hidalgo *et al.*, 2009; Sánchez-Valle *et al.*, 2020). A disease pair i and j was defined as having EHR-driven comorbidity when both measures indicated a tendency to co-occur ($\phi_{ij}$ > 0 and ${\mathrm{RR}}_{\mathrm{ij}}$ > 1).

Taking as ground truths the corresponding EHR-driven comorbidity, DDN-driven comorbidity scores f from Equation (5) for each unlabeled disease can be decomposed into $f^{+}$ and $f^{-}$ based on a threshold value. Suppose that $\hat{y}={\left({\hat{y}}_{1},\;\ldots ,{\hat{y}}_{m}\right)}^{T}$ is the ground truths, then ${\hat{y}}_{i}$ can take a value of ‘+1’ if disease i is defined as having EHR-driven comorbidity, and ‘−1’ otherwise. Then, the receiver operating characteristic (ROC) curve was analyzed to determine a threshold value J for deciding the labels on f. Youden’s J statistic was used for a threshold value where the maximum value of J=sensitivity+specificity-1, as in a ROC curve (Schisterman *et al.*, 2005). We categorized diseases as having DDN-driven direct comorbidity (high chance of co-occurrence) when $f^{+}\;=\;\left\{f_{i}\;\right|\;f_{i}\geq J,\;\mathrm{for}\;\forall i\}$ and DDN-driven inverse comorbidity (low chance of co-occurrence) when$\;f^{-}\;=\;\left\{f_{i}\;\right|\;f_{i}<J,\;\mathrm{for}\;\forall i\}$.

## 3 Results

### 3.1 Data processing for network construction and validation

**Fig. 3.**
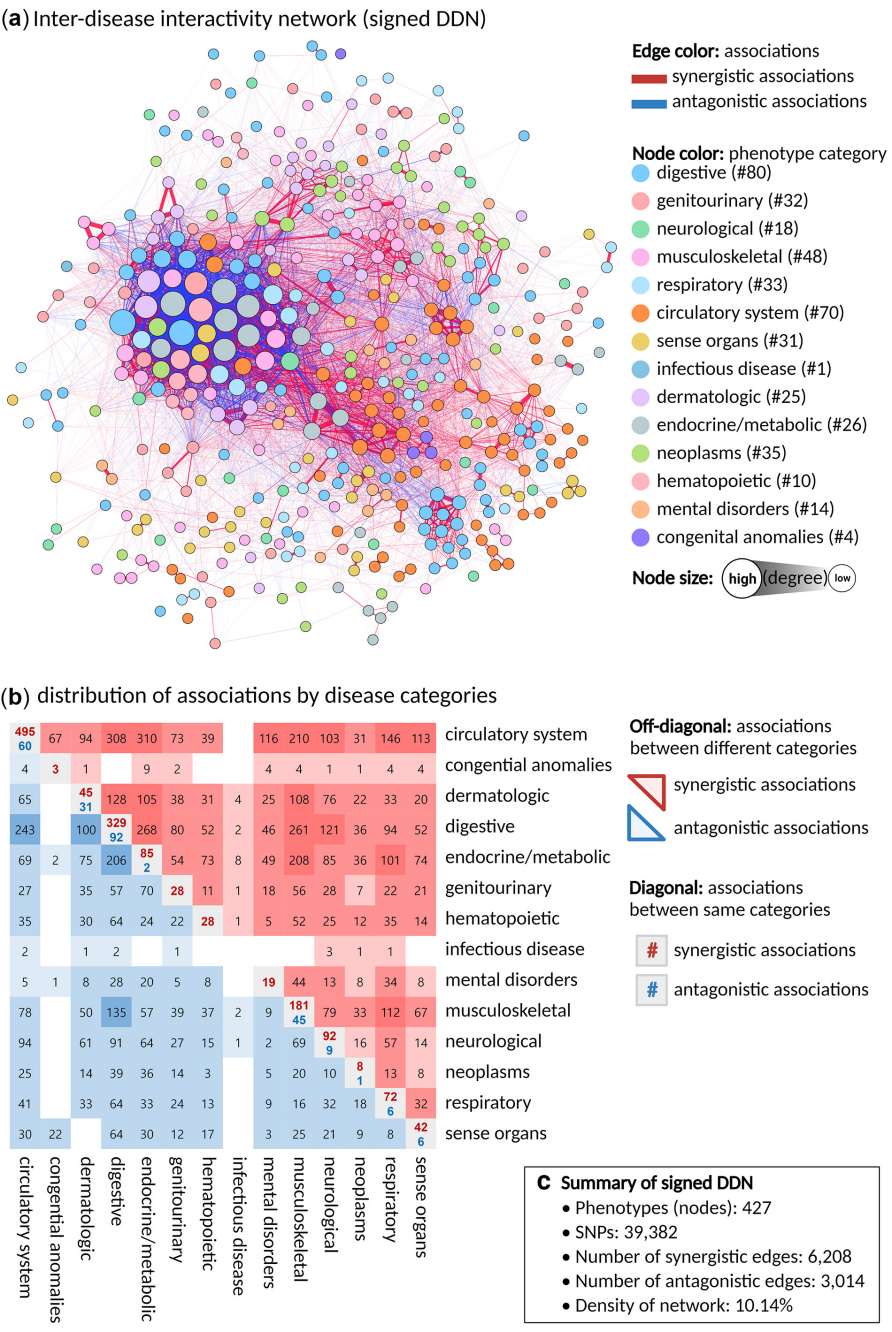
Visualization of an inter-disease interactivity network (signed DDN). (**a**) A signed DDN with 427 phenotypes. Nodes represent diseases, and edges represent the shared SNP associations between pairs of diseases. Node color indicates phenotype category, and size the degree of connections. Each line indicates either synergistic (positive values, Supplementary Fig. S1) or antagonistic (negative values, Supplementary Fig. S2) associations between nodes. Supplementary Figure S3 shows heatmap for signed DDN (**b**) distribution of disease associations among the 14 phenotype categories. Upper and lower diagonal elements, respectively, depict the numbers of positive and negative associations across different categories. The main diagonal represents associations among diseases belonging to the same phenotype category. (**c**) Summarized information on the data used for constructing the signed DDN

Disease–SNP associations were generated from UK Biobank PheWAS summary statistics based on EHR-derived broad phenotype codes (PheCode) (Wei *et al.*, 2017; Wu *et al.*, 2019). The PheWAS summary statistics were obtained from https://www.leelabsg.org/resources, and include 1403 phenotypes (Zhou *et al.*, 2018), of which 976 were excluded in this study due to having a relatively low number of cases (<1000) or representing injuries and poisonings, symptoms, or sex-specific disease. Ultimately, network construction and comorbidity prediction were performed on 427 diseases belonging to 14 phenotype categories (Fig. 3). Pre-processing of the PheWAS summary results was performed using the following steps: (i) 588 711 independent SNPs were selected by linkage disequilibrium pruning with a threshold (window size: 50 kb, step size: 5 kb, and *r*^2^ threshold: 0.5), and (ii) SNPs with association *P*-value < 1 × 10^−4^ were selected, yielding 39 382 SNPs for analysis. Disease–SNP associations were represented as a (427 × 39 382)-dimensional matrix in which the association of each element pair was represented by a *z*-score. This matrix was then used to construct a DDN with signed associations using Equation (1).

EHR-driven comorbidity information was collected and generated from the UK Biobank hospital episode statistic database. Disease diagnoses for 502 505 UK Biobank participants are represented by ICD-9 and ICD-10 codes, which were mapped to PheCodes by referring to PheCode Map versions 1.2 and 1.2b1 (https://phewascatalog.org/). A patient–phenotype matrix was constructed with binary values indicating the diagnosis status of each participant for 427 diseases to calculate ϕ-correlation and relative risk. More details about the UK Biobank PheWAS results are provided in Supplementary Text S1, and the phenotypes used in this analysis are listed in Supplementary Data S1.

### 3.2 Construction of the inter-disease interactivity network (signed DDN)

The resulting inter-disease interactivity network (Fig. 3a) consisted of 427 phenotypes (nodes) and 9223 total associations (edges), of which 6209 were synergistic (67%; red) and 3014 antagonistic (33%; blue) (Fig. 3c and Supplementary Figs S1 and S2). We examined the distribution of associations across the 14 phenotype categories (Fig. 3b), and found connections between diseases within a phenotype category to typically be synergistic. Namely, diseases belonging to a given phenotype category often had consistent effect directions for their shared SNPs, which means that the chance of their co-occurrence may be higher. One feature of signed DDNs is the ability to observe genetic associations between diseases as the direction of effect of shared SNPs. Our signed DDN was constructed while taking into account both the total amount and direction of shared SNPs. The greater the number of shared SNPs, the stronger the association between two diseases; likewise, the more homogenous the direction of effect, the stronger the synergistic association. We examined the composition of synergistic/antagonistic associations in our signed DDN. Of the 427 diseases in the network, 364 feature both associations, 57 have only synergistic associations, 3 have only antagonistic associations and, remaining 3 diseases have no associations (Supplementary Data S2, https://hdpm.biomedinfolab.com/ddn/signedDDN). To investigate a deeper biological interpretations of the network, we described the composition of phenotypes and interactions in signed DDN for coronary atherosclerosis (PheCode: 411.4) as case example (Supplementary Text S2). For example, Coronary atherosclerosis and type 2 diabetes (PheCode: 250.2) shared 66 SNPs, of which 55 SNPs were associated with positive direction of effects and 11 with negative direction of effects in relation to both diseases. By calculating the overall direction of effects (similarity = 0.29), coronary atherosclerosis and type 2 diabetes were determined to have a synergistic association.

### 3.3 Comparison of comorbidity prediction performance

The objective of network-based comorbidity scoring algorithms using a signed DDN is to predict whether other diseases are more or less likely to co-occur when one disease occurs. We hypothesized that the direction of effects of shared SNP is informative in predicting comorbidity and in prioritizing disease co-occurrence. Namely, higher scores indicate greater sharing of SNPs and more consistent direction of SNP effects; thus, diseases having stronger synergistic association with a given index disease have higher predicted comorbidity scores. To test this hypothesis, we compared prediction performance between the proposed method (signed DDN) and the baseline method (unsigned DDN). The unsigned DDN was constructed as in previous studies (Goh *et al.*, 2007; Nam *et al.*, 2019a; Verma *et al.*, 2019), with only the number of shared genetic components being considered. The difference between signed and unsigned DDNs is explained in Supplementary Figure S4. A transductive approach was used, in which only one set of experiments was carried per disease. EHR-driven comorbidity was used for ground truths. Prediction performance was evaluated in terms of the area under the receiver operating characteristics curve (AUC) and Spearman rank correlation (*ρ*) (Fawcett, 2006; Yule, 1919). Both AUC and rank correlation for an index disease of interest were obtained by comparing the predicted DDN-driven comorbidity scores and EHR-driven comorbidity scores (Supplementary Fig. S5).

**Fig. 4.**
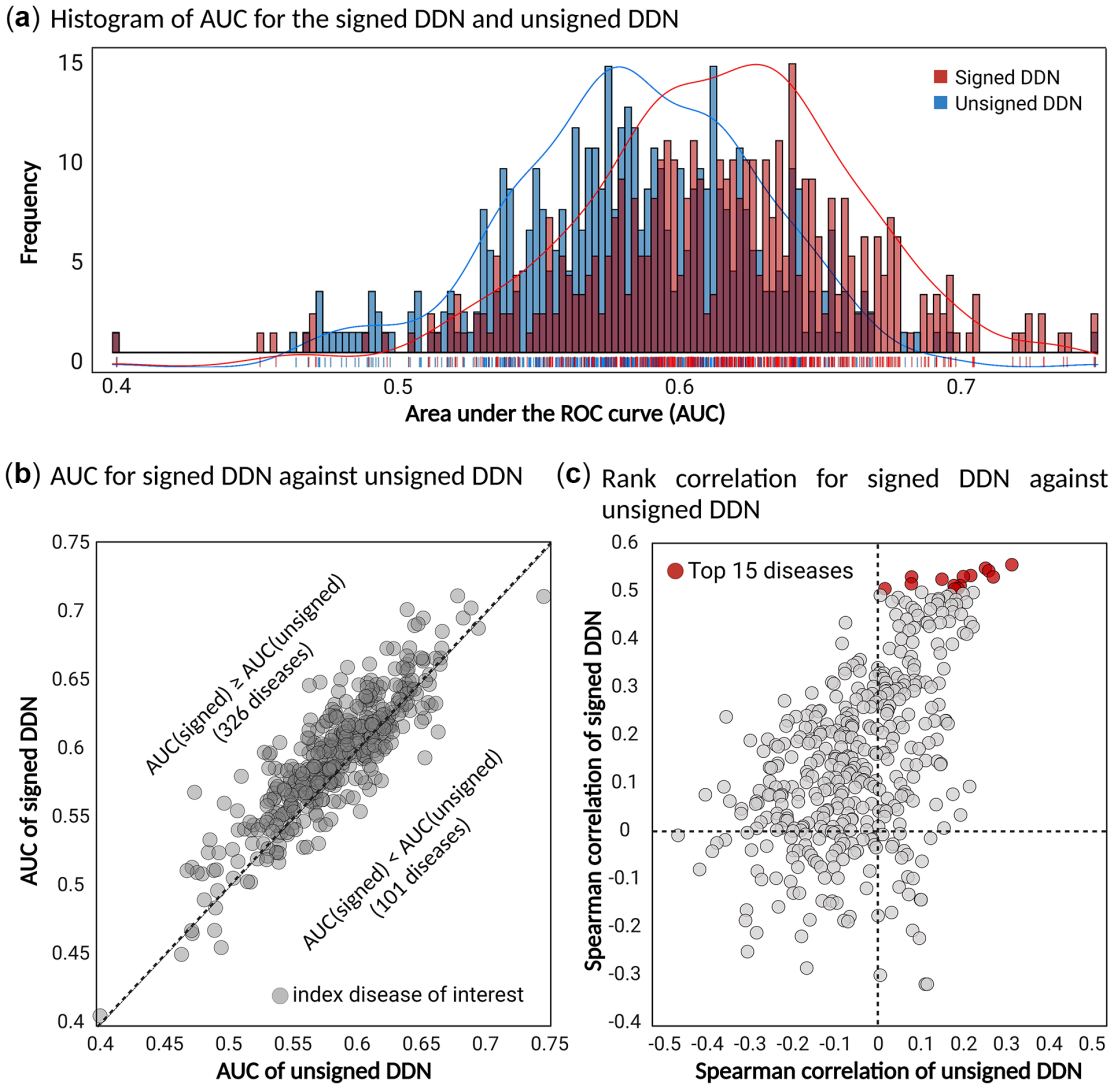
Improvement of performance with the proposed signed DDN in comparison to an unsigned network. (**a**) Histogram of AUC values for the signed and unsigned DDNs. AUC values are binned along the *x*-axis. The bars depict the disease frequency in each bin for both networks. The histogram is shifted towards right for the signed DDN. (**b**) Plot of signed DDN AUC against that of the unsigned DDN. The *y*- and *x*-axis indicate AUC values for the signed and unsigned networks, respectively. Each circle indicates a set of experiments for one disease. (**c**) Comparison of Spearman rank correlations. Spearman rank correlations indicate ordinal associations between rankings of DDN-comorbidity scores (*f*) and rankings of EHR-driven relative risks

Comparison of prediction performance between the signed and unsigned DDNs revealed that the interaction of the association between phenotypes can be helpful in predicting comorbidity (Fig. 4). The AUC distribution of the 427 phenotypes in the signed DDN was shifted higher relative to that of the unsigned DDN (Fig. 4a). Moreover, the signed DDN demonstrated superior performance, achieving an average of 0.601 (versus 0.573 with the unsigned DDN) and with values generally falling above the diagonal when plotted against corresponding unsigned scores (Fig. 4b). In the same manner, we compared Spearman rank correlation coefficients between the two DDNs (Fig. 4c). In the signed DDN, 341 diseases had positive correlations (above zero on *y*-axis, Fig. 4c), of which 259 exhibited significant correlations (0.103–0.556, *P*-value < 0.05). In the unsigned DDN, 163 diseases had positive correlations (above zero on *x*-axis, Fig. 4c), of which 53 were statistically significant (0.105–0.290, *P*-value < 0.05). Only five diseases statistically significant in the unsigned DDN achieved higher positive correlation coefficients than were determined with the signed DDN. Thus, direct comparison of DDN-comorbidity score and prevalence-based relative risk indicates that taking into account the direction of effect of shared SNPs could better predict disease comorbidity. Notably, when not considering direction of effect, only weakly positive or non-significant correlations were obtained between prevalence-based relative risk and predicted scores. Both AUCs and Spearman rank correlation coefficients can be interpreted as the explanatory power of comorbidity predictions for index diseases. Performance measurement can be affected by the statistical power of PheWAS for each disease, but they are affected more greatly by the structure of the network because they are the predicted result through a scoring algorithm. Thus, an index disease with a high AUC means that disease associations explained by genetic information can be interpreted as comorbidity relationships in epidemiology.

### 3.4 Interpretation of the correlation coefficient in signed/unsigned DDNs

**Fig. 5.**
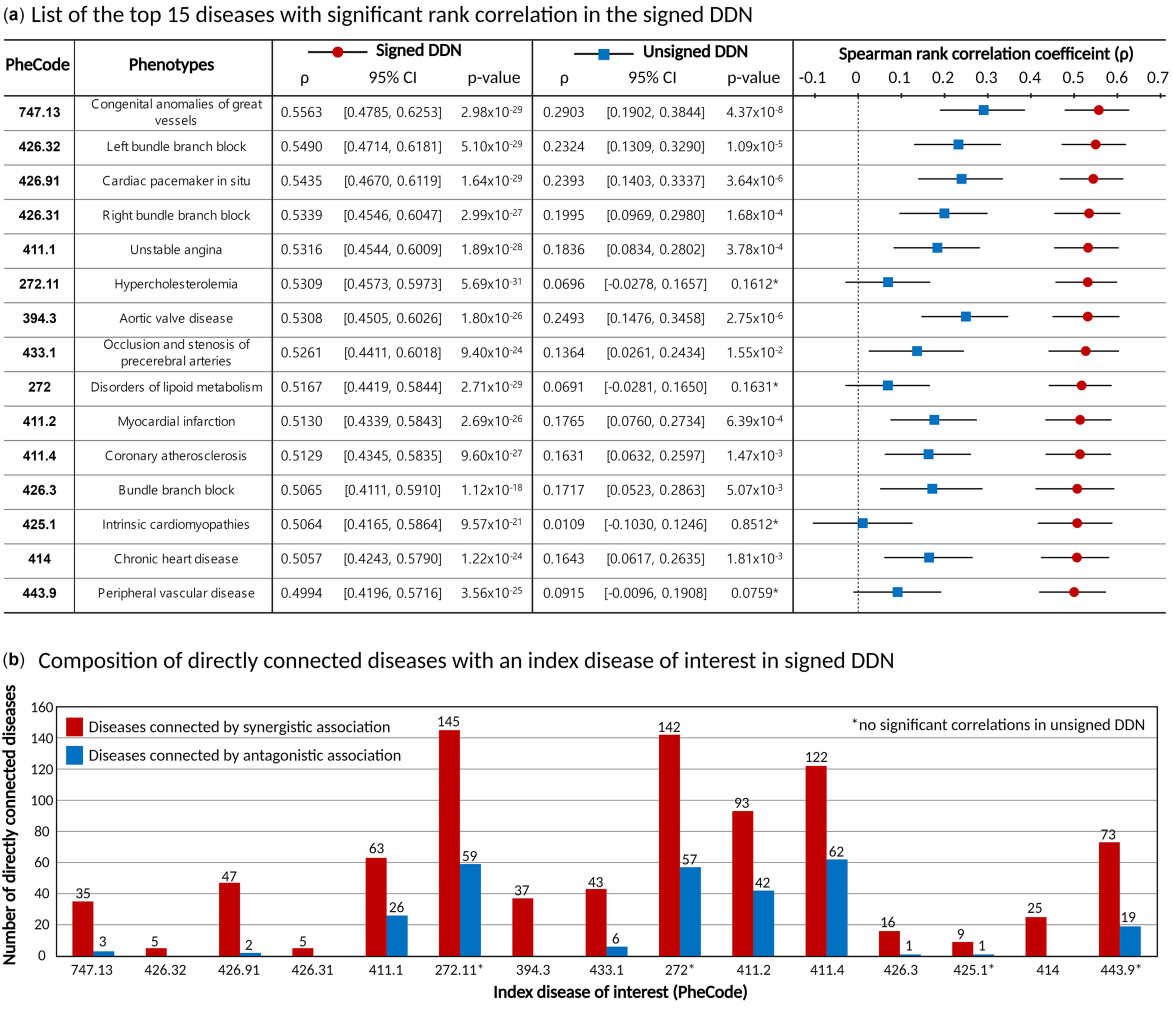
List of the top 15 diseases with significant rank correlations in the signed DDN. (**a**) Spearman rank correlation in the signed DDN against the unsigned DDN. Diseases were sorted in descending order with respect to correlation coefficient in signed DDN. (**b**) Composition of directly connected diseases. Red bars indicate the number of synergistic associations and blue bars the number of antagonistic associations among diseases directly connected with the given index disease. Four diseases with a superscript in *P*-value were not statistically significant in the unsigned DDN

We further investigated the top 15 diseases with significant rank correlations (marked as red in Fig. 4c). DDN-comorbidity scores for these diseases in the signed DDN were highly correlated with prevalence-based relative risk (*ρ* > 0.5 with *P*-value < 0.05), whereas those in the unsigned DDN were relatively low (0 < *ρ* < 0.3). Overall, the top 15 included 11 circulatory-related diseases that had high positive correlation coefficients in the signed DDN (Fig. 5a). Four diseases with highly significant correlations in the signed DDN did not achieve significance in the unsigned DDN (superscript in *P*-value, Fig. 5a). It can be inferred that considering SNP direction of effect and its commonality between diseases is capable of capturing complex disease associations that might not be identified when only considering sharing of SNPs.

In this approach, synergistically associated diseases directly connected to the index disease are likely to have the highest predicted scores. Consequently, we examined which diseases had direct connections when taking each of the 15 top diseases as the index disease (Fig. 5b). The results showed all diseases with high correlation coefficients to have relatively high ratios of synergistic association. In addition, we found that comorbidity scores derived from the signed DDN were highly correlated with EHR-driven comorbidity, even for diseases having many antagonistic associations, such as hypercholesterolemia, disorders of lipoid metabolism, myocardial infarction and coronary atherosclerosis. This further supports that considering the direction of effects is helpful when using PheWAS data to analyze the complex relationships between diseases, as the latent associations between diseases (synergistic/antagonistic association) provide important information for comorbidity prediction.

### 3.5 Clinical implication of comorbidity scoring based on the signed DDN

**Fig. 6.**
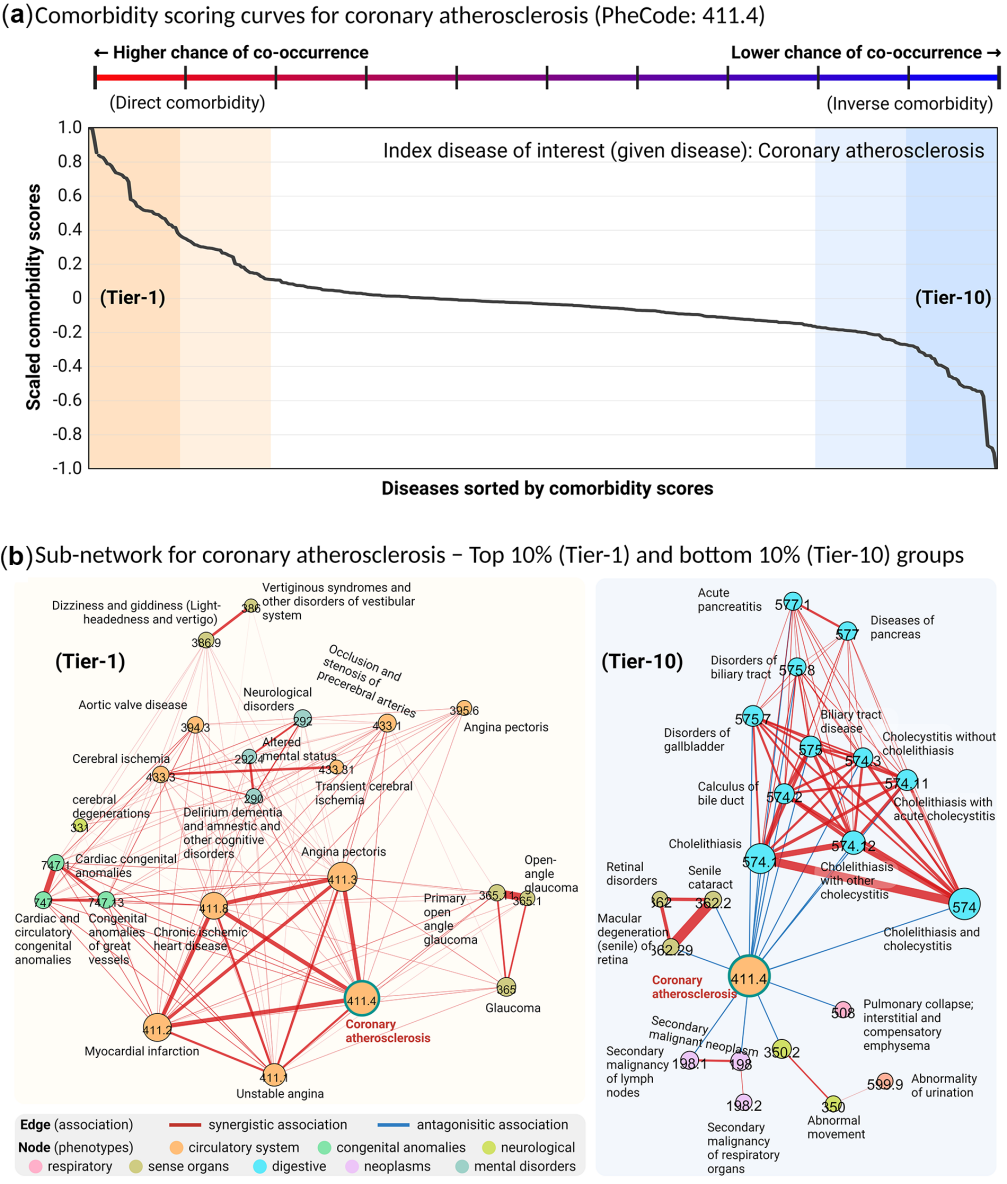
Comorbidity scoring curves and associations for coronary atherosclerosis. (**a**) Comorbidity scoring curves for coronary atherosclerosis (PheCode: 411.4). The solid line indicates DDN-driven comorbidity scores (*f*) of the other 426 diseases in the dataset, sorted in descending order with respect to score on the *x*-axis. The *y*-axis represents comorbidity scores, rescaled to values between −1 and 1. (**b**) Sub-networks of diseases having high comorbidity (Tier-1) and low comorbidity (Tier-10) with coronary atherosclerosis

We demonstrated how to predict disease comorbidity by applying a graph-based SSL for signed graph to a signed DDN encompassing 427 diseases. To provide a case study illustrating the proposed method and its interpretation, coronary atherosclerosis was selected from among the top 15 diseases having significant rank correlations in the signed DDN. Coronary atherosclerosis is a frequent cause of coronary artery disease, a common chronic condition for which disease risk is characterized by a substantial and complex polygenic contribution, with heritability between 40% and 60% (Fischer *et al.*, 2005; Marenberg *et al.*, 1994; McPherson and Tybjaerg-Hansen, 2016). We provided the comorbidity scores for a list of direct comorbidity diseases (Table 1), and the comorbidity score curve obtained from Equation (5) (Fig. 6a). To suggest the practical use of comorbidity scores, we divided the scores into deciles and categorized accordingly, ranging from high risk of comorbidity (direct comorbidity) to low risk of comorbidity (inverse comorbidity). We decided the Tier-1 diseases as direct comorbidity group and Tier-10 diseases as inverse comorbidity group according to their ranking of predicted scores. The remaining diseases between Tier-2 and Tier-9 were relatively lower co-occurrence than Tier-1 or higher co-occurrence than Tier-10.

**Table 1.** The top 20 diseases having direct comorbidity with coronary atherosclerosis

Phenotypes (PheCode)	Scores	RR [95% CI]	ϕ -corr
Myocardial infarction (411.2)	1.000	13.052 [13.050, 13.054]	0.5564
Other chronic ischemic heart disease, unspecified (411.8)	0.993	11.636 [11.634, 11.637]	0.5521
Angina pectoris (411.3)	0.929	11.510 [11.508, 11.511]	0.5573
Unstable angina (411.1)	0.855	13.261 [13.256, 13.266]	0.3512
Cardiac congenital anomalies (747.1)	0.840	4.115 [4.000, 4.234]	0.0185
Cardiac and circulatory congenital anomalies (747)*	0.831	0.918 [0.344, 2.445]	−0.0002
Congenital anomalies of great vessels (747.13)	0.827	8.758 [8.746, 8.770]	0.1439
Other cerebral degenerations (331)	0.811	2.432 [2.348, 2.519]	0.0100
Primary open angle glaucoma (365.11)	0.790	1.620 [1.601, 1.639]	0.0092
Open-angle glaucoma (365)	0.788	1.614 [1.595, 1.632]	0.0091
Delirium dementia and amnestic and other cognitive disorders (290)	0.777	3.311 [3.261, 3.361]	0.0211
Altered mental status (292.4)	0.756	2.880 [2.871, 2.889]	0.0400
Dizziness and giddiness (386.9)	0.738	3.114 [3.110, 3.119]	0.0624
Heart valve replaced (395.6)	0.734	9.291 [9.276, 9.306]	0.1371
Aortic valve disease (394.3)	0.729	7.573 [7.557, 7.590]	0.1042
Glaucoma (365)	0.724	1.882 [1.876, 1.888]	0.0234
Cerebral ischemia (433.3)	0.710	3.670 [3.650, 3.690]	0.0385
Occlusion and stenosis of precerebral arteries (433.1)	0.706	6.338 [6.316, 6.361]	0.0718
Vertiginous syndromes and other disorders of vestibular system (386)	0.684	2.008 [1.681, 2.400]	0.0034
Neurological disorders (292)	0.580	2.782 [2.741, 2.825]	0.0177

*Note*: An asterisk denotes a disease pair that were predicted as having direct comorbidity from DDN-driven comorbidity, but inverse comorbidity on the basis of EHR-driven comorbidity (RR, relative risk; 95% CI, 95% confidence interval; corr, correlation).

We examined the sub-networks for diseases (Fig. 6b) having high risk of co-occurrence (Tier-1) and those having low risk of co-occurrence (Tier-10). All Tier-1 diseases were synergistically associated with coronary atherosclerosis and also with each other, which means that high-ranked diseases overall have shared SNPs with consistent direction of effect. In contrast, within the Tier-10 group, all diseases directly connected with coronary atherosclerosis had antagonistic associations and the predicted scores were negative. In Tier-10 group, macular degeneration (PheCode: 362.29) was antagonistically associated with coronary atherosclerosis and the predicted comorbidity was inverse. The scoring algorithm with the signed DDN detected the known relationships that high levels of HDL-C were associated with low risk of coronary artery disease and associated with high risk of age-related macular degeneration (Fan *et al.*, 2017). A sub-network consisting of all diseases directly connected with coronary atherosclerosis is given in Supplementary Figure S6. The top 20 diseases having direct comorbidity with coronary atherosclerosis are summarized in Table 1.

### 3.6 Criterion of leveraging disease–SNP associations in summary statistics

We developed a signed DDN by leveraging the overall direction of the effects of genetic components shared between diseases. We also performed predictions of co-occurrence diseases given an index disease to examine whether the signed DDN can have more reliable explanatory power for disease interactions. To propagate the seed label information of index disease to the rest of the diseases in a network through the scoring algorithm, we took a less stringent genome-wide significance level (*P*-value < 1 × 10^−4^) for network construction. However, depending on the arbitrary selection of significance level, the underlying structure of the network can be changed. The disease interactions are sparser at the most stringent level and denser at the less stringent level. It is necessary to explore which *P*-value threshold in the disease–SNP associations for defining disease associations. We built another comparative DDN by performing LDSC regression to investigate whether comorbidity prediction results varied according to the number of SNPs in building a network. We built the genetic correlation-based DDN (denoted as LDSC-DDN) by performing LDSC regression with UK Biobank PheWAS summary data to estimate the genetic correlations of 427 pairs used for the signed DDN (Bulik-Sullivan *et al.*, 2015). The LD scores were calculated from European samples in the 1000 Genomes Project phase 3 database (Auton *et al.*, 2015). We considered disease pairs with positive and negative correlation values with significance (*P*-value < 0.05). We took the correlation matrix as the similarity matrix of the DDN and applied the proposed scoring algorithm to predict comorbidity. The LDSC-DDN had a higher density value of 19.97% (18 165 edges across 427 nodes) than the signed DDN (density of 10.14% as shown in Fig. 3c). We performed comorbidity prediction tasks and calculated AUCs with the same experimental settings as the signed network. Empirically, the dense network can provide more accurate inferences than sparse network. However, comorbidity prediction for 98 diseases could not be conducted because they were disconnected from other diseases in the LDSC-DDN. The LDSC results cannot be obtained when applied to datasets with low statistical power of GWAS (due to small sample size or rare trait) for estimating heritability and genetic correlation. Most connections in the LDSC-DDN were obtained from diseases with high statistical power. The proposed signed DDN had advantage of discovering inter-disease interactions by leveraging significantly associated SNPs, even though the number of SNPs used for constructing network was smaller. The detailed results are provided in Supplementary Text S3.

## 4 Discussion and conclusion

We proposed a novel signed DDN based on biobank-scale PheWAS summary statistics that considers the direction of effect of shared SNPs, and further presented the utility of this DDN in prioritizing diseases according to comorbidity. To design this DDN, we measured the overall direction of effect of SNPs shared between pairs of diseases, and further categorized disease–disease associations as synergistic or antagonistic depending on whether that overall direction is consistent or opposite between diseases. Our results demonstrated that considering the interaction and direction of shared components is more helpful in predicting comorbidity and ranking comorbid diseases than considering only the quantity of shared components.

We also developed a novel label propagation algorithm for signed networks and applied it to show that the signed DDN not only models disease relationships at the population level, but also is applicable to comorbidity prediction in the context of personalized medicine. The comorbidity scoring algorithm was designed such that when the initial label on the index disease encounters a ‘disease connected by a synergistic association’, the score is propagated with a positive value (increasing the likelihood of co-occurrence), whereas when it encounters a ‘disease connected by an antagonistic association’, the score is propagated with a negative value (reducing the likelihood of co-occurrence).

One advantage of the proposed method is that the signed DDN can aid in the interpretation and comprehension of the intricate associations among diseases, and moreover can be easily updated and reinforced when new PheWAS results are obtained for a phenotype not yet included in the network. A second advantage is its ability to categorize diseases as having direct and inverse comorbidity based on the defined synergistic/antagonistic associations. It is very important to know both which conditions have higher chance of co-occurrence (direct comorbidity) and which have lower chance of co-occurrence (inverse comorbidity) for a given disease. Such categorized relationships enable clinicians to construct comorbidity scenarios in advance, and can aid patient treatment planning. Our study also used co-occurrence and disease prevalence information obtained from UK Biobank hospitalization episodes to confirm and validate the DDN-driven predictions of direct/inverse comorbidity. Although omnidirectional validation has not been performed, our findings support that a DDN based on the relations of diseases and genetic components provides sufficient information for the prioritization and categorization of disease comorbidity.

Some aspects of this study remain to be investigated in future work. One of the primary limitations is that we utilized data from a single cohort of the UK Biobank. Since the UK Biobank mainly includes healthy participants, the constructed network might not capture disease prevalence in populations of different demographic characteristics. We also limited our analyses to those phenotypes occurring in both sexes. Some significant genetic components might vary by sex, and thus it is necessary to construct sex-specific signed DDNs and determine sex-specific comorbidity. Although our stratification of comorbidity groups is a product of a specific population, the approach can be extended for use in precision medicine to screen individuals at comorbidity risks.

## Supplementary Material

btac822_Supplementary_Data

## Data Availability

The UK Biobank summary data are publicly available at https://www.leelabsg.org/resources. The source codes and data are available on https://github.com/dokyoonkimlab/DiseaseInteractiveNetwork, the implementation of inter-disease interactivity network is available on https://hdpm.biomedinfolab.com/ddn/signedDDN.

## References

[btac822-B1] Auton A. et al; 1000 Genomes Project Consortium. (2015) A global reference for human genetic variation. Nature, 526, 68–74. 26432245 10.1038/nature15393 PMC4750478

[btac822-B2] Barabási A.-L. et al (2011) Network medicine: a network-based approach to human disease. Nat. Rev. Genet., 12, 56–68. 21164525 10.1038/nrg2918 PMC3140052

[btac822-B3] Buddeke J. et al (2019) Comorbidity in patients with cardiovascular disease in primary care: a cohort study with routine healthcare data. Br. J. Gen. Pract., 69, e398–e406. 31064742 10.3399/bjgp19X702725 PMC6532812

[btac822-B4] Bulik-Sullivan B. et al; Genetic Consortium for Anorexia Nervosa of the Wellcome Trust Case Control Consortium 3. (2015) An atlas of genetic correlations across human diseases and traits. Nat. Genet., 47, 1236–1241. 26414676 10.1038/ng.3406 PMC4797329

[btac822-B5] Catalá-López F. et al (2014) Inverse and direct cancer comorbidity in people with central nervous system disorders: a meta-analysis of cancer incidence in 577,013 participants of 50 observational studies. Psychother. Psychosom., 83, 89–105. 24458030 10.1159/000356498

[btac822-B6] Cho S.I. et al (2021) Impact of comorbidity burden on mortality in patients with COVID-19 using the Korean health insurance database. Sci. Rep., 11, 1–9. 33737679 10.1038/s41598-021-85813-2 PMC7973767

[btac822-B7] Chong Y. et al (2020) Graph-based semi-supervised learning: a review. Neurocomputing, 408, 216–230.

[btac822-B8] Darabos C. et al (2015) A bipartite network approach to inferring interactions between environmental exposures and human diseases. Pac. Symp. Biocomput., **2015**, 171–182. 25592579

[btac822-B9] Di Angelantonio E. et al; Emerging Risk Factors Collaboration. (2009) Major lipids, apolipoproteins, and risk of vascular disease. JAMA, 302, 1993–2000. 19903920 10.1001/jama.2009.1619 PMC3284229

[btac822-B10] Dong G. et al (2021) A global overview of genetically interpretable multimorbidities among common diseases in the UK Biobank. Genome Med., 13, 110. 34225788 10.1186/s13073-021-00927-6 PMC8258962

[btac822-B11] Fan Q. et al (2017) HDL-cholesterol levels and risk of age-related macular degeneration: a multiethnic genetic study using Mendelian randomization. Int. J. Epidemiol., 46, 1891–1902. 29025108 10.1093/ije/dyx189 PMC5837540

[btac822-B12] Fawcett T. (2006) An introduction to ROC analysis. Pattern Recognit. Lett., 27, 861–874.

[btac822-B13] Fischer M. et al (2005) Distinct heritable patterns of angiographic coronary artery disease in families with myocardial infarction. Circulation, 111, 855–862. 15710764 10.1161/01.CIR.0000155611.41961.BB

[btac822-B14] Gallier J. (2016) Spectral theory of unsigned and signed graphs. Applications to graph clustering: a survey. arXiv:1601.04692.

[btac822-B15] Goh K.-I. et al (2007) The human disease network. Proc. Natl. Acad. Sci. USA, 104, 8685–8690. 17502601 10.1073/pnas.0701361104 PMC1885563

[btac822-B16] Hidalgo C.A. et al (2009) A dynamic network approach for the study of human phenotypes. PLoS Comput. Biol., 5, e1000353. 19360091 10.1371/journal.pcbi.1000353 PMC2661364

[btac822-B17] Ibáñez K. et al (2014) Molecular evidence for the inverse comorbidity between central nervous system disorders and cancers detected by transcriptomic meta-analyses. PLoS Genet., 10, e1004173. 24586201 10.1371/journal.pgen.1004173 PMC3930576

[btac822-B18] Jørgensen T.L. et al (2012) Comorbidity in elderly cancer patients in relation to overall and cancer-specific mortality. Br. J. Cancer, 106, 1353–1360. 22353805 10.1038/bjc.2012.46 PMC3314782

[btac822-B19] Klimek P. et al (2015) Quantification of diabetes comorbidity risks across life using nation-wide big claims data. PLoS Comput. Biol., 11, e1004125. 25855969 10.1371/journal.pcbi.1004125 PMC4391714

[btac822-B20] Lee D.-G. et al (2020) Dementia key gene identification with multi-layered SNP-gene-disease network. Bioinformatics, 36, i831–i839. 33381851 10.1093/bioinformatics/btaa814

[btac822-B21] Lee D.S. et al (2008) The implications of human metabolic network topology for disease comorbidity. Proc. Natl. Acad. Sci. USA, 105, 9880–9885. 18599447 10.1073/pnas.0802208105 PMC2481357

[btac822-B22] Liu C.C. et al (2014) DiseaseConnect: a comprehensive web server for mechanism-based disease-disease connections. Nucleic Acids Res., 42, W137–W146. 24895436 10.1093/nar/gku412 PMC4086092

[btac822-B23] Marenberg M.E. et al (1994) Genetic susceptibility to death from coronary heart disease in a study of twins. N. Engl. J. Med., 330, 1041–1046. 8127331 10.1056/NEJM199404143301503

[btac822-B24] McPherson R. , Tybjaerg-Hansen A. (2016) Genetics of coronary artery disease. Circ. Res., 118, 564–578. 26892958 10.1161/CIRCRESAHA.115.306566

[btac822-B25] Nam Y. et al (2019a) Disease gene identification based on generic and disease-specific genome networks. Bioinformatics, 35, 1923–1930. 30335143 10.1093/bioinformatics/bty882

[btac822-B26] Nam Y. et al (2019b) The translational network for metabolic disease–from protein interaction to disease co-occurrence. BMC Bioinformatics, 20, 12. 31722666 10.1186/s12859-019-3106-9 PMC6854734

[btac822-B27] Nam Y. et al (2022) netCRS: network-based comorbidity risk score for prediction of myocardial infarction using biobank-scaled PheWAS data. Pac. Symp. Biocomput., 27, 325–336. 34890160 PMC8682919

[btac822-B28] Nashiry A. et al (2021) Bioinformatics and system biology approach to identify the influences of COVID-19 on cardiovascular and hypertensive comorbidities. Brief. Bioinform., 22, 1387–1401. 33458761 10.1093/bib/bbaa426 PMC7929376

[btac822-B29] Richardson S. et al; the Northwell COVID-19 Research Consortium. (2020) Presenting characteristics, comorbidities, and outcomes among 5700 patients hospitalized with COVID-19 in the New York city area. JAMA, 323, 2052–2059. 32320003 10.1001/jama.2020.6775 PMC7177629

[btac822-B30] Roitmann E. et al (2014) Patient stratification and identification of adverse event correlations in the space of 1190 drug related adverse events. Front. Physiol., 5, 332. 25249979 10.3389/fphys.2014.00332 PMC4158870

[btac822-B31] Rubio-Perez C. et al (2017) Genetic and functional characterization of disease associations explains comorbidity. Sci. Rep., 7, 1–14. 28740175 10.1038/s41598-017-04939-4 PMC5524755

[btac822-B32] Sánchez-Valle J. et al (2020) Interpreting molecular similarity between patients as a determinant of disease comorbidity relationships. Nat. Commun., 11, 2854. 32504002 10.1038/s41467-020-16540-x PMC7275044

[btac822-B33] Schisterman E.F. et al (2005) Optimal cut-point and its corresponding Youden Index to discriminate individuals using pooled blood samples. Epidemiology, 16, 73–81. 15613948 10.1097/01.ede.0000147512.81966.ba

[btac822-B34] Sharrett A.R. et al; Atherosclerosis Risk in Communities Study Group. (2001) Coronary heart disease prediction from lipoprotein cholesterol levels, triglycerides, lipoprotein(a), apolipoproteins A-I and B, and HDL density subfractions: the atherosclerosis risk in communities (ARIC) study. Circulation, 104, 1108–1113. 11535564 10.1161/hc3501.095214

[btac822-B35] Subramanya A. , Talukdar P.P. (2014) Graph-based semi-supervised learning. Synth. Lect. Artif. Intell. Mach. Learn., 8, 1–125.

[btac822-B36] Tabarés-Seisdedos R. , Baudot A. (2016) Direct and inverse comorbidities between complex disorders. Front. Physiol., 7, 117. 27065885 10.3389/fphys.2016.00117 PMC4811936

[btac822-B37] Tabarés-Seisdedos R. , Valderas J.M. (2013) Inverse comorbidity: the power of paradox in the advancement of science. J. Comorb., 3, 1–3. 29090139 10.15256/joc.2013.3.19 PMC5636022

[btac822-B38] Tarantino G. et al (2018) Prediction of carotid intima-media thickness in obese patients with low prevalence of comorbidities by serum copper bioavailability. J. Gastroenterol. Hepatol., 33, 1511–1517. 29405466 10.1111/jgh.14104

[btac822-B39] Valderas J.M. et al (2009) Defining comorbidity: implications for understanding health and health services. Ann. Fam. Med., 7, 357–363. 19597174 10.1370/afm.983 PMC2713155

[btac822-B40] Verma A. et al; DiscovEHR Collaboration. (2019) Human-disease phenotype map derived from PheWAS across 38,682 individuals. Am. J. Hum. Genet., 104, 55–64. 30598166 10.1016/j.ajhg.2018.11.006 PMC6323551

[btac822-B41] von Mutius E. , Smits H.H. (2020) Primary prevention of asthma: from risk and protective factors to targeted strategies for prevention. Lancet, 396, 854–866. 32910907 10.1016/S0140-6736(20)31861-4

[btac822-B42] Wei W.-Q. et al (2017) Evaluating phecodes, clinical classification software, and ICD-9-CM codes for phenome-wide association studies in the electronic health record. PLoS One, 12, e0175508. 28686612 10.1371/journal.pone.0175508 PMC5501393

[btac822-B43] Wu P. et al (2019) Mapping ICD-10 and ICD-10-CM codes to phecodes: workflow development and initial evaluation. JMIR Med. Inform., 7, e14325. 31553307 10.2196/14325 PMC6911227

[btac822-B44] Yule G.U. (1919) An Introduction to the Theory of Statistics. C. Griffin, Limited, London, UK.

[btac822-B45] Zheng J. et al (2018) PhenoSpD: an integrated toolkit for phenotypic correlation estimation and multiple testing correction using GWAS summary statistics. Gigascience, 7, giy090. 30165448 10.1093/gigascience/giy090 PMC6109640

[btac822-B46] Zheng J. et al; Early Genetics and Lifecourse Epidemiology (EAGLE) Eczema Consortium. (2017) LD Hub: a centralized database and web interface to perform LD score regression that maximizes the potential of summary level GWAS data for SNP heritability and genetic correlation analysis. Bioinformatics, 33, 272–279. 27663502 10.1093/bioinformatics/btw613 PMC5542030

[btac822-B47] Zhou W. et al (2018) Efficiently controlling for case-control imbalance and sample relatedness in large-scale genetic association studies. Nat. Genet., 50, 1335–1341. 30104761 10.1038/s41588-018-0184-y PMC6119127

[btac822-B48] Zhou X. et al (2014) Human symptoms-disease network. Nat. Commun., 5, 4212. 24967666 10.1038/ncomms5212

[btac822-B49] Zhu Z. et al (2018) Causal associations between risk factors and common diseases inferred from GWAS summary data. Nat. Commun., 9, 12. 29335400 10.1038/s41467-017-02317-2 PMC5768719

