# Supplementary material for: Discovering comorbid diseases using an inter-disease interactivity network based on biobank-scale PheWAS data
Source: Bioinformatics. 2022 Dec 26;39(1):btac822. doi: 10.1093/bioinformatics/btac822 (PMC9825330; doi:10.1093/bioinformatics/btac822)
Supplement: btac822_Supplementary_Data [file btac822_supplementary_data.zip › Supplementary_File S1_revised_221128.docx]

**Supplementary Text**

**Supplementary Text S1.**

**Description of UK biobank PheWAS results**

**UK Biobank study and data disclosure statement** UK Biobank is a prospective cohort of over 500,000 participants aged 40 to 69 years when recruited 2006 to 2010, that contains genetic information linked to electronic health records (EHR) and is one of the largest repositories. The UK biobank is also included 531 diagnostic terms and the hospitalization episode statistics data that utilize 10^th^ revision of the International Classification of Diseases (ICD) codes and contain 16,310 diagnostic terms (https://biobank.ndph.ox.ac.uk/ukb/). The UK Biobank has obtained ethical approval covering this study from the National Research Ethics Committee (reference no. 11/NW/0382); extended on May 10, 2016 [RES reference 16/NW/0274]). The UK Biobank dataset was obtained from the UK Biobank (Application Number 33002), and a full list of the variables are available online. Data cannot be shared publicly due to the violation of patient privacy and the absence of informed consent for data sharing.

**UK Biobank PheWAS summary statistics** We used SAIGE UK Biobank summary statistics based on EHR-derived broad PheCodes^1,2^. This SAIGE approach is feasible for genetic association studies in large-scale Biobanks by accounting for sample relatedness^3^. Thus, this approach prevents excessive sample dropout by genetic quality control, resulting in more reliable SNPs based on larger samples for constructing disease-disease networks. A total of 427 PheWAS summary statistics that considered in our network include genetic association results on binary outcomes for 28 million variants with counts ranging from 1,002 to 77,723 cases and 330,366 to 407,809 controls in white British.

**UK Biobank hospital episode statistics data.** We collected EHR-driven comorbidity information based on EHR-derived broad PheCodes using UK Biobanks’ HES data. PheCode is an effective term that maps clinically meaningful phenotypes many-to-one for PheWAS [ref 1,2]. Of a total of 1746 PheCodes, 976 were used that are mapped to ICD-9 and ICD-10 diagnosed. We configured the Parents’ code (e.g. PheCode 411) to cover the Childs’ codes (e.g. PheCode 411.1, 411.2, 411.3, 411.41, 411.5, 411.8, and 411.9). For PheCode mapping for ICD-9 and ICD-10, we referred to Phecode Map 1.2 and Phecode Map 1.2b1 to ICD-10 (https://phewascatalog.org/), respectively.

**Reference**

[1] Wei, Wei-Qi, et al. "Evaluating phecodes, clinical classification software, and ICD-9-CM codes for phenome-wide association studies in the electronic health record." *PloS one***12.7** (2017): e0175508.

[2] Wu, Patrick, et al. "Mapping ICD-10 and ICD-10-CM codes to phecodes: workflow development and initial evaluation." *JMIR medical informatics***7.4** (2019): e14325.

[3] Zhou, Wei, et al. "Efficiently controlling for case-control imbalance and sample relatedness in large-scale genetic association studies." *Nature genetics* **50.9** (2018): 1335-1341.

**Supplementary Text S2.**

**Composition of phenotypes and associations in the signed DDN**

We examined the composition of synergistic/antagonistic associations in our signed DDN. Of the 427 diseases in the network, 364 feature both associations, 57 have only synergistic associations, 3 have only antagonistic associations, and remaining three diseases have no associations (Supplementary Data S2, <https://hdpm.biomedinfolab.com/ddn/signedDDN>). For example, coronary atherosclerosis (PheCode: 411.4) was connected with a total of 184 diseases in either 122 synergistic or 62 antagonistic associations. Coronary atherosclerosis and type 2 diabetes (PheCode: 250.2) shared 66 SNPs (Fig. 1a), of which 55 SNPs were associated with positive direction of effects and 11 with negative direction of effects in relation to both diseases. By calculating the overall direction of effects (similarity = 0.29; see Methods), coronary atherosclerosis and type 2 diabetes were determined to have a synergistic association. Coronary atherosclerosis was also connected with asthma (PheCode: 495) in a synergistic association (similarity = 0.04) governed by 48 significant shared SNPs (Fig. 1b), of which 45 had the same direction of effect and two (rs72938315 and rs2033784) had opposite directions of effect. As the calculated overall direction of effect for these 48 shared SNPs was positive, the diseases have a synergistic association. In a third example, coronary atherosclerosis and macular degeneration (PheCode: 362.29) have an antagonistic association determined by shared 2 SNPs (rs17649495 and rs9349507) with opposite direction of effects; positive effect for coronary atherosclerosis and negative effect for macular degeneration (Fig. 1c). Coronary atherosclerosis and cholelithiasis (PheCode: 574.1) have an antagonistic association (similarity = -0.0015) because their shared SNPs include nine with opposite direction of effect and one with positive direction of effect (rs3130279, Fig. 1d).

**
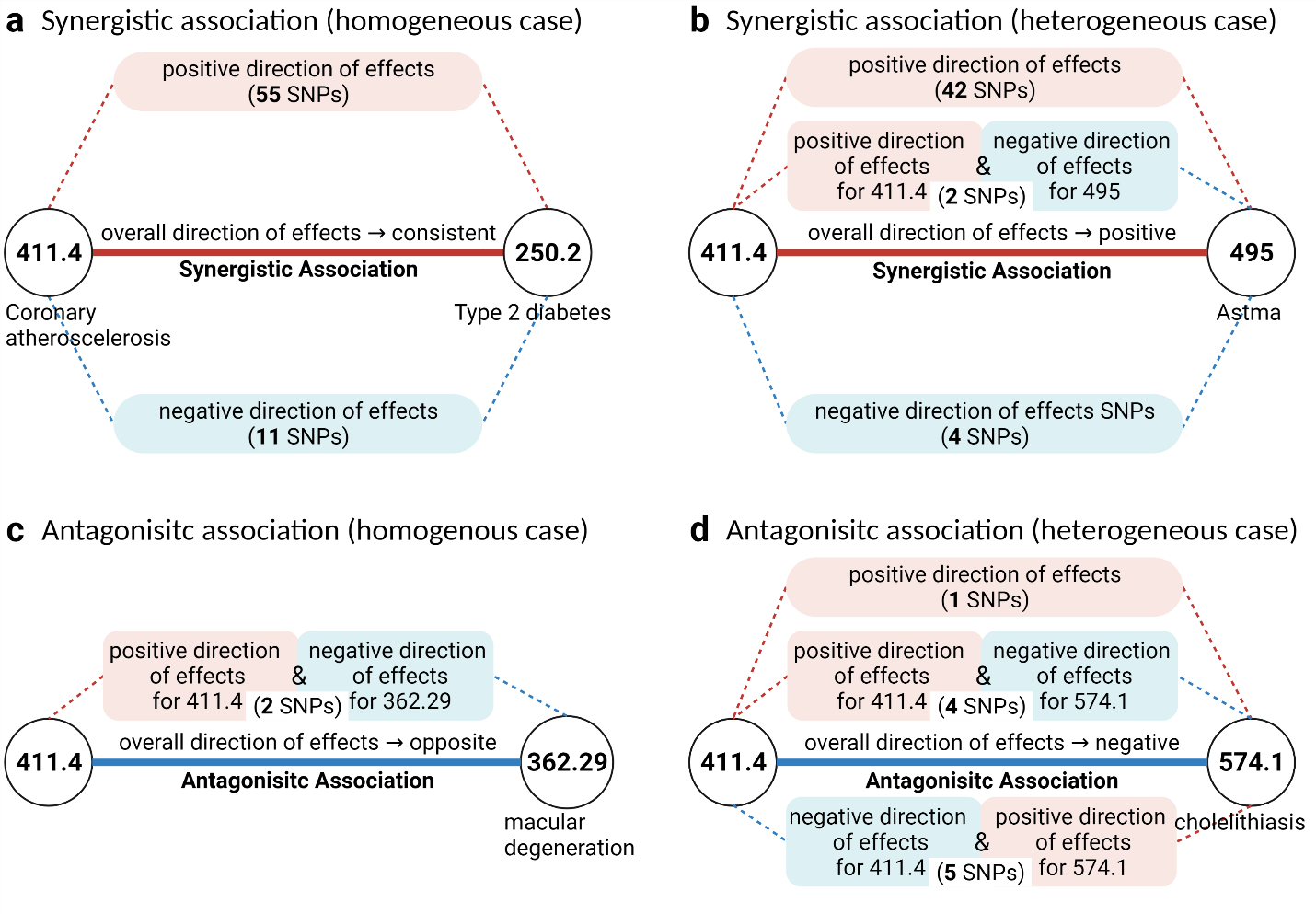
**

**Fig. 1. Visualization of direction of effect among SNPs shared between coronary atherosclerosis and other diseases.** Synergistic and antagonistic associations were calculated by Eq. (1) in Methods. Detailed list of shared SNPs between two diseases were described in Fig. 2 in manuscript.

**
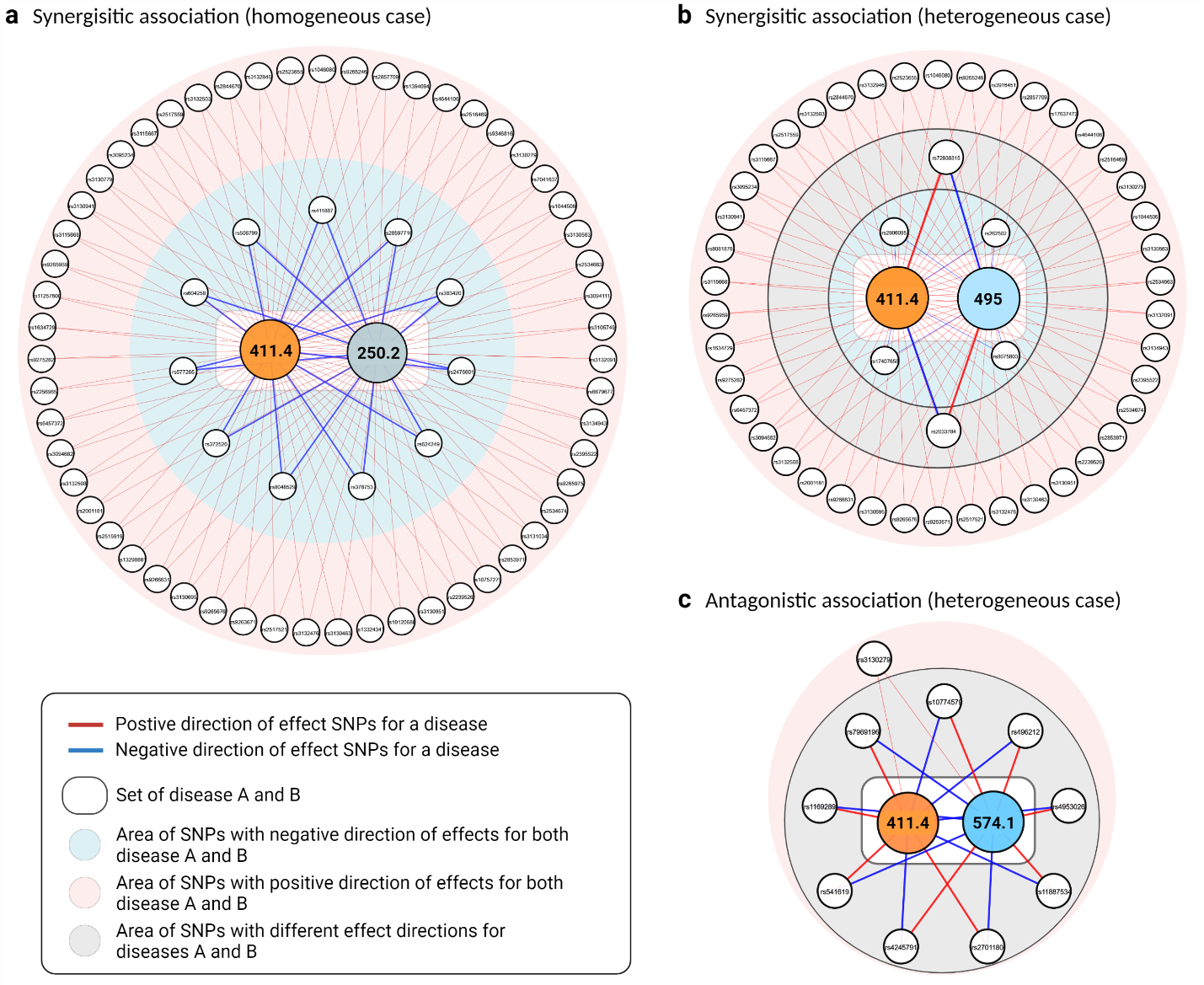
Determining disease associations into synergistic or antagonistic**

**Fig. 2. Example cases for determining disease associations between coronary atherosclerosis (PheCode: 411.4) and other disease by considering direction of effect among SNPs. a) Synergistic association with homogenous direction of effects between diseases and SNPs.** The coronary atherosclerosis and type 2 diabetes (PheCode: 250.2) have shared 66 SNPs. 55 SNPs (in red region) were positive direction of effects and 11 SNPs (in blue region) were negative direction of effects between both diseases. Since all SNPs are in the same direction for both diseases regardless of direction, the two nodes are connected by synergistic association. **b) Synergistic associations with heterogeneous direction of effects.** Two diseases have shared 48 SNPs; 42 SNPs were positively associated and 4 SNPs were negatively associated with both diseases. 2 SNPs (in gray region) were positively associated with coronary atherosclerosis and were negatively associated with asthma (PheCode: 495). When calculating overall directions (see Methods), the overall direction was positive. **c) Antagonistic associations with heterogeneous direction of effects.** Coronary atherosclerosis and cholelithiasis (PheCode: 574.1) have an antagonistic association because their shared SNPs include nine with opposite direction of effect (gray region) and one with positive direction of effect (rs3130279, red region)

**Supplementary Text S3.**

**Predicting comorbidity using LDSC-based DDN**

We performed linkage disequilibrium score (LDSC) regression to estimate the genetic correlations of 427 pairs using UK Biobank PheWAS summary data used for the proposed DDN construction. The LD scores were calculated from European samples in the 1000 Genomes Project phase 3 database. We considered disease pairs with positive and negative correlation values with significance (p-value < 0.05). We constructed LDSC-based DDN using the calculated genetic correlations between diseases. The LDSC-based DDN and the proposed signed DDN differ in the extent of observing disease-associated SNPs to define connections between diseases. Although LDSC using the entire SNPs is likely to observe latent disease-disease associations, possible false-positive associations in the PheWAS summary may affect the disease-disease associations. To investigate the impact on accounting the number of SNPs in summary statistics, we performed the comorbidity prediction experiments. We calculated AUCs with the same experimental settings as the signed network. Empirically, the dense network can provide more accurate inferences than the sparse network.


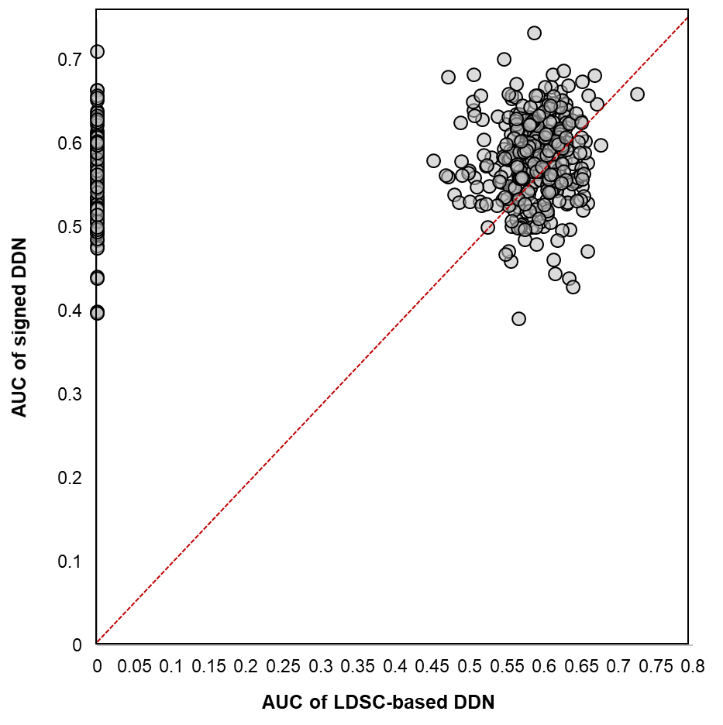
However, comorbidity prediction for 98 diseases could not be conducted because they were disconnected from other diseases in the LDSC-DDN. The LDSC results cannot be obtained when applied to datasets with low statistical power of GWAS summary (due to small sample size or rare trait) for estimating heritability and genetic correlation. Most connections in the LDSC-DDN were obtained from diseases with high statistical power. The proposed signed DDN had advantage of discovering inter-disease interactions by leveraging significantly associated SNPs, even though the number of SNPs used for constructing network was smaller and their statistical power of GWAS summary was low. The summarized AUCs are described in the following Figure.

**Figure. Comparison of prediction performance between LDSC-DDN and signed DDNSupplementary Algorithm**

| **INPUT:**   1. **Collected PheWAS Summary data**   $S^{\mathrm{SNP}}$: Set of unique SNPs in total PheWAS summary data  $S^{\mathrm{Disease}}$: Set of phenotype-wise association vectors in PheWAS summary data   1. **Collected clinical records**   $C_{\mathrm{ij}}$: Number of patients who had both diseases *i* and *j*  $P_{i}$: Number of patients with disease *i*  N: Total number of patients in clinical records   1. **Hyper-parameter**   $\alpha$: thresholds for selecting significance of SNPs in PheWAS data  $\mu$: Hyper-parameter for graph-based SSL  **PROCEDURE**:   1. **Constructing signed disease-disease network**   Generating disease – SNPs association matrix $\boldsymbol{R}$ from PheWAS data  $R_{ik}=\left\{ \begin{matrix} \frac{\beta_{ik}}{SE_{ik}} & \mathrm{if} \mathrm{diseas}e_{i} \sim\mathrm{SN}P_{k} has associations consdiering p-\mathrm{value}<\alpha\\ 0 & \mathrm{otherwise} \end{matrix} \right.$,  Calculating disease-disease association **W**  $w_{ij}=\frac{\sum_{k\in S} (r_{ik})\cdot(r_{jk})}{\sqrt{\sum_{k=1}^{K} r_{ik}^{2}}\sqrt{\sum_{k=1}^{K} r_{jk}^{2}}}$, where $S$ is set of shared SNPs in both disease *i* and *j*   1. **Predicting comorbidity scores**   Calculating signed diagonal degree matrix: $\bar{D}=diag(\bar{d}_{i})$ where $\bar{d_{i}}=\sum_{j} \vert w_{ij}\vert$  Calculating signed Graph Laplacian $\bar{L}=\bar{D}-W$  **For** $\mathbf{i=1:\vert}\mathbf{S}^{\mathbf{Disease}}\mathbf{\vert}$ Calculating scores for each disease  Initializing labeled set: $\boldsymbol{y}^{\boldsymbol{(}i\boldsymbol{)}}={(0, \ldots,0,y_{i}=1, 0,\ldots,0)}^{T}$  Applying scoring function from (5): $\boldsymbol{f}^{\boldsymbol{(}i\boldsymbol{)}}=\left( \boldsymbol{I}+\mu\bar{\boldsymbol{L}} \right)^{-1}\boldsymbol{y}^{\boldsymbol{(}i\boldsymbol{)}}$  Calculating clinical record-based comorbidity as ground truth for label decision  **For** $\mathbf{j=1:\vert}\mathbf{S}^{\mathbf{Disease}}\mathbf{\vert}$  Calculating relative risk and phi-correlation: $\mathrm{RR}_{\mathrm{ij}}$, $\phi_{\mathrm{ij}}$  Clinical-based comorbidity: $\boldsymbol{z}^{\boldsymbol{(i)}}=\left\{ \begin{matrix} +1 & \mathrm{if} RR_{ij}>1, \phi_{ij}>0 \\ -1 & otherwise \end{matrix} \right.$  **End for j**  Deciding candidate direct and inverse comorbidity  Calculating ROC: $\left[ fpr, tpr, th \right]\leftarrow\mathrm{ROC}(f^{\left( i \right)}, z^{\left( i \right)})$  where $\mathrm{fpr}$ is false-positive rate, $\mathrm{tpr}$ is true positive rate, and $\mathrm{th}$ is thresholds  Find Youden index J: $j^{*}\boldsymbol{\leftarrow}\underset{\mathrm{th}}{\mathbf{argmax}} J\left( \mathrm{th} \right)=Sen\left( \mathrm{th} \right)+Spec\left( \mathrm{th} \right)-1$  Deciding candidate direct comorbidity: $\boldsymbol{f}^{\left( i \right)+}=\left\{ \boldsymbol{f}^{\left( i \right)} \right\vert\boldsymbol{f}^{(i)}\geq j^{*})$  Deciding candidate inverse comorbidity $\boldsymbol{f}^{\left( i \right)-}=\left\{ \boldsymbol{f}^{\left( i \right)} \right\vert\boldsymbol{f}^{(i)}<j^{*})$  **End for**  **OUTPUT:**   1. **Signed disease-disease network:** $\mathbf{G=(}\mathbf{S}^{\mathbf{Disease}}\boldsymbol{, W)}$ 2. **Network-based comorbidity scores:** $\boldsymbol{f=}\left[ \boldsymbol{f}^{\left( \boldsymbol{1} \right)}\boldsymbol{;}\boldsymbol{f}^{\left( \boldsymbol{2} \right)}\boldsymbol{\ldots;}\boldsymbol{f}^{\left( \left\vert\boldsymbol{S}^{\boldsymbol{Disease}} \right\vert\right)} \right]$ 3. **Candidate direct and inverse comorbidity lists:** $\boldsymbol{f}^{\boldsymbol{+}}$**,** $\boldsymbol{f}^{\boldsymbol{-}}$ |
| --- |

**Algorithm 1. Pseudo-code for network-based comorbidity scoring algorithms**

**Supplementary Figures**

**
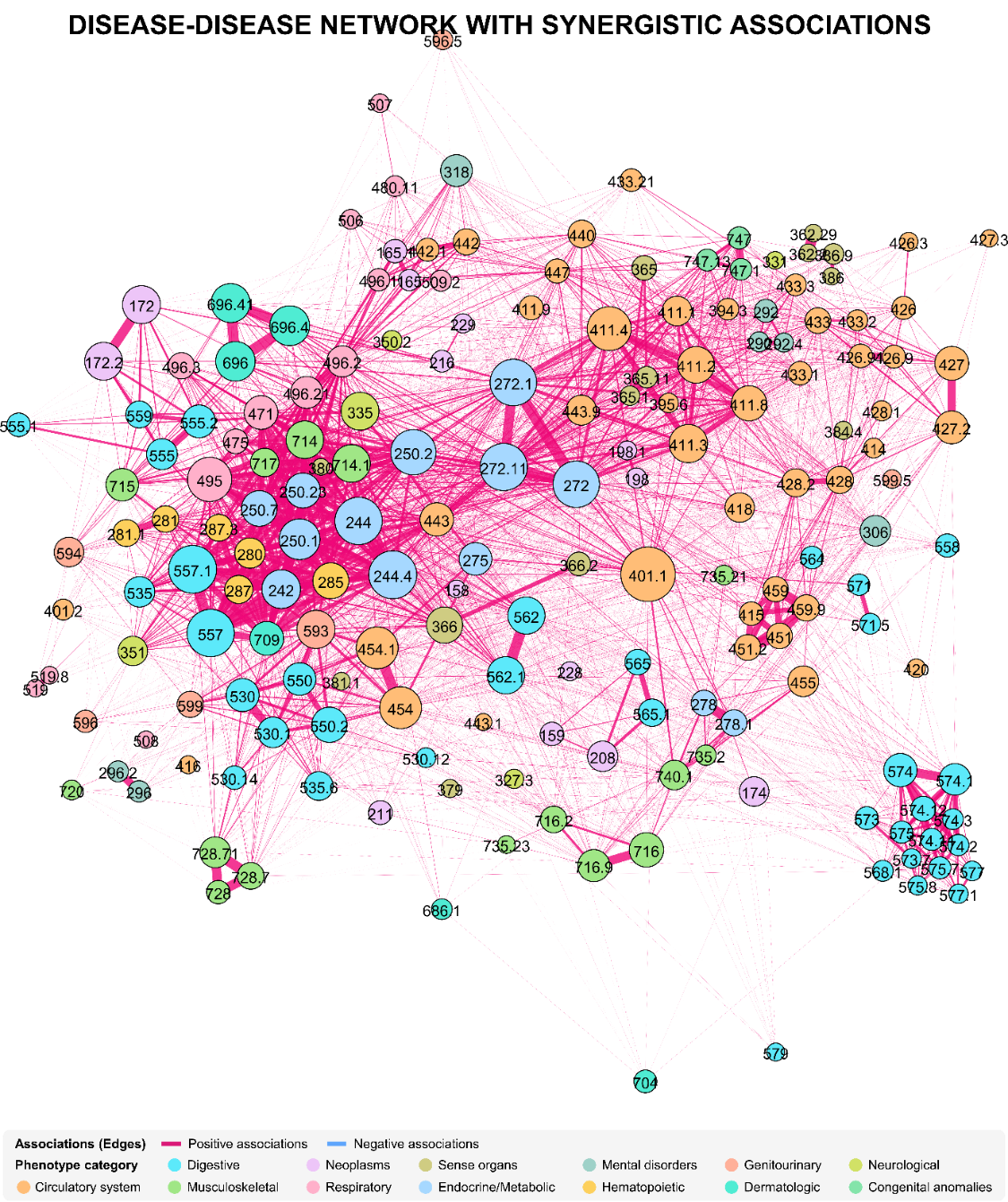
Supplementary Figure S1.**

**Figure S1. Composed singed DDN with only synergistic associations.** The full signed DDN with both associations were described in figure 2a. Since the associations between two diseases have either synergistic or antagonistic, we can decomposed signed DDN into signed DDN with synergistic and antagonistic associations. 427 phenotypes were connected with 6,209 synergistic associations.

**
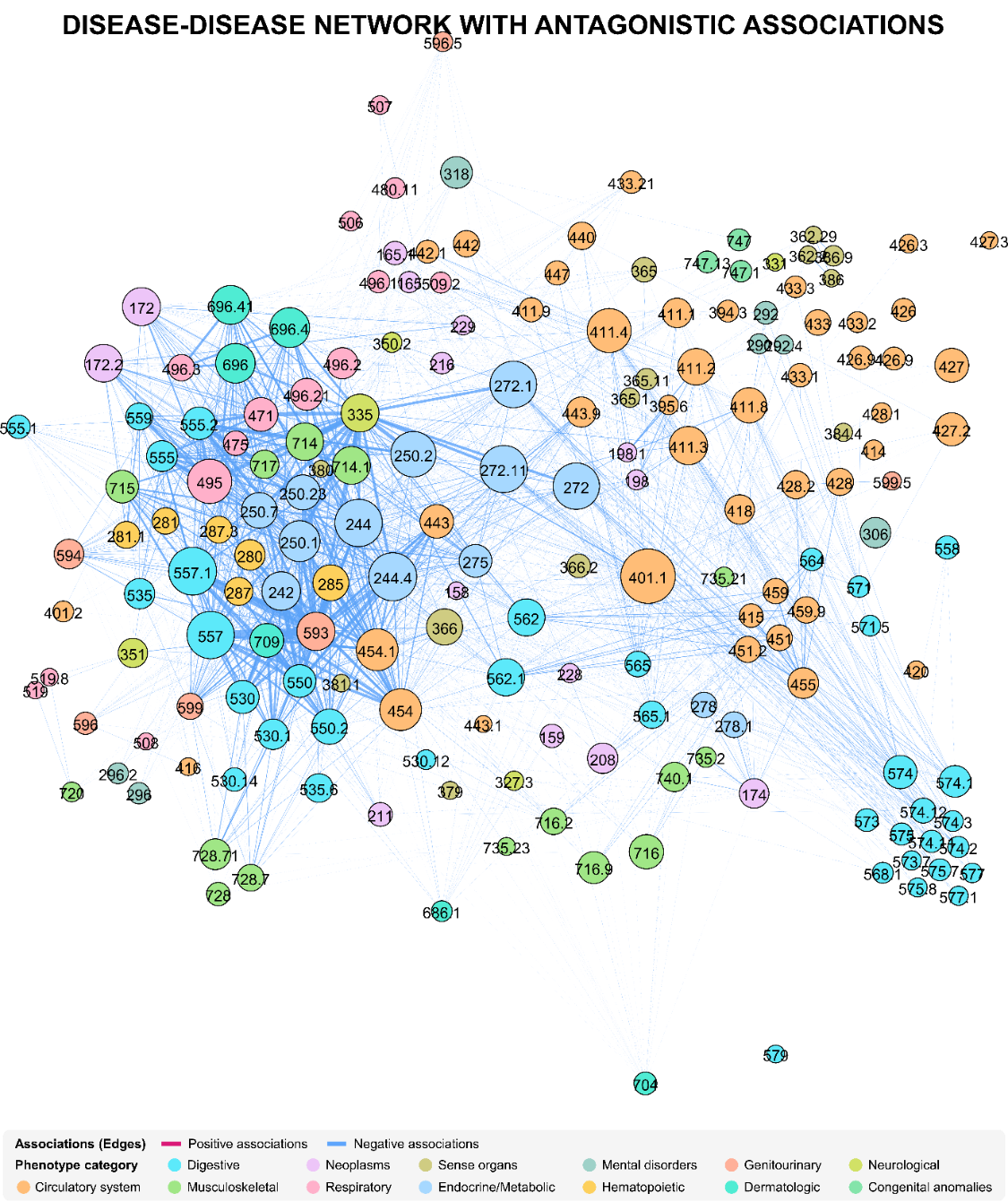
Supplementary Figure S2.**

**Figure S2. Composed singed DDN with only antagonistic associations.** The full signed DDN with both associations were described in figure 2a. Since the associations between two diseases have either synergistic or antagonistic, we can decomposed signed DDN into signed DDN with synergistic and antagonistic associations. 427 phenotypes were connected with 3,014 antagonistic associations.

**Supplementary Figure S3.**

**Figure S3. Heatmap for signed DDN.
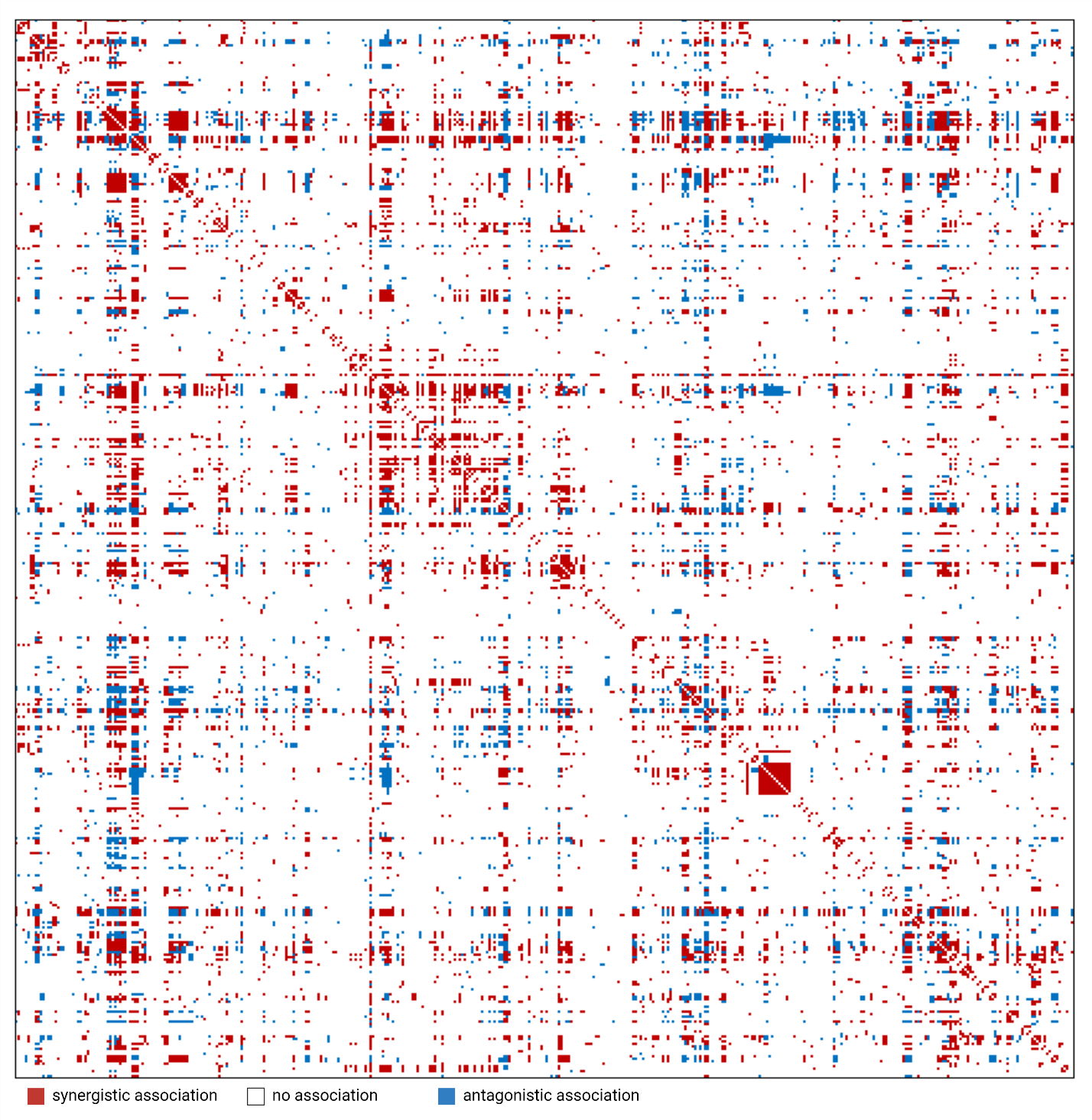
**

**
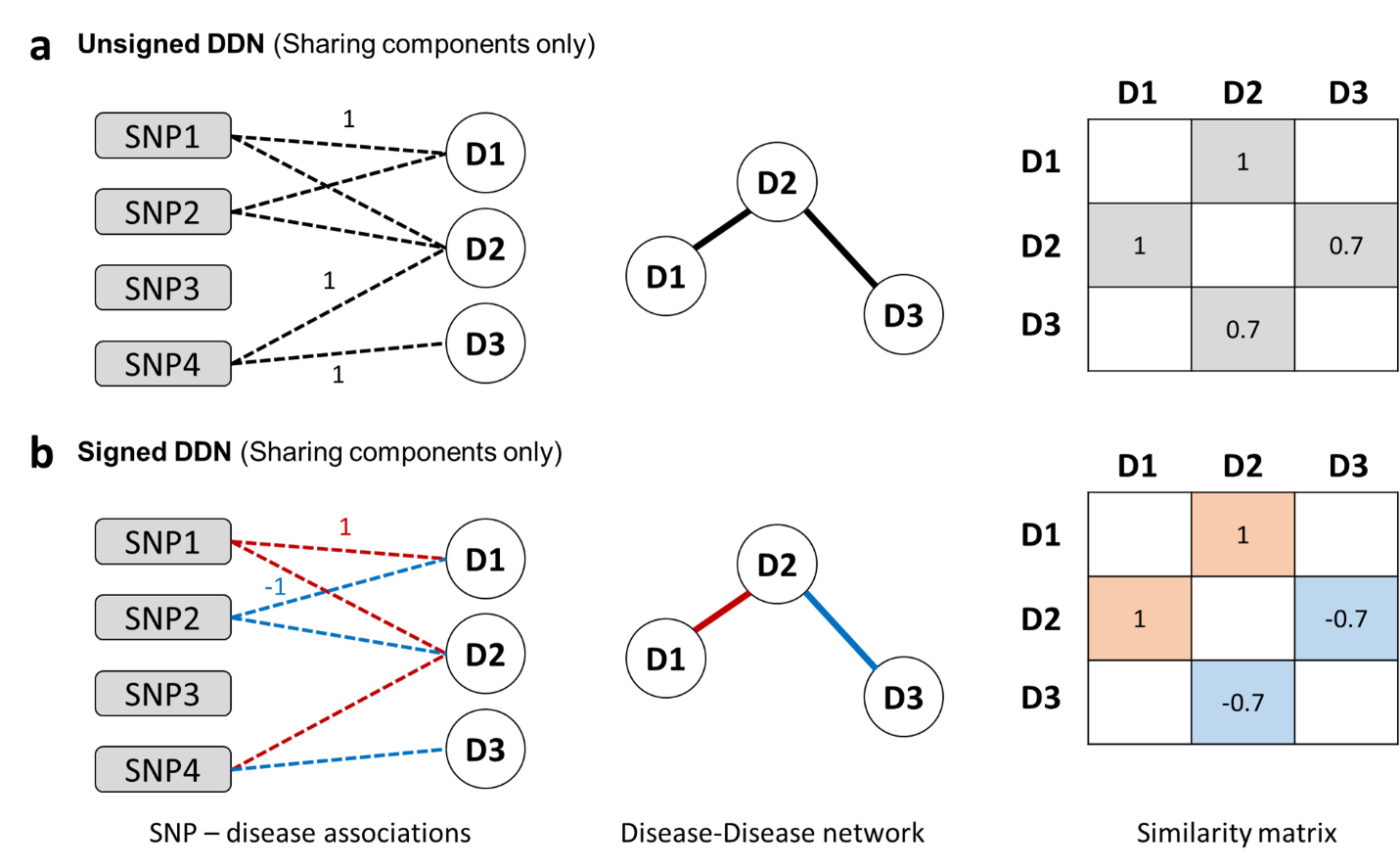
Supplementary Figure S4. Difference between singed and unsigned DDN**

**Figure S4. Difference between signed and unsigned DDN. a) Unsigned DDN:** Edges were only considered presence(‘1’) or absence of associated SNPs. Edge weight represents the number of shared SNPs. **b) Signed DDN:** Considering original z-scores between disease and SNPs from PheWAS summary. Here, to explain easily, we assume the all z-scores for disease-SNPs association is ‘1’ (positive value, positive direction of effects) or ‘-1’ (negative value, negative direction of effects). By accounting directions of effects and the number of shared SNPs using cosine similarity, we can obtain disease-disease network with opposite signs. In similarity matrix, positive value means synergistic associations between diseases and negative value means antagonistic association.


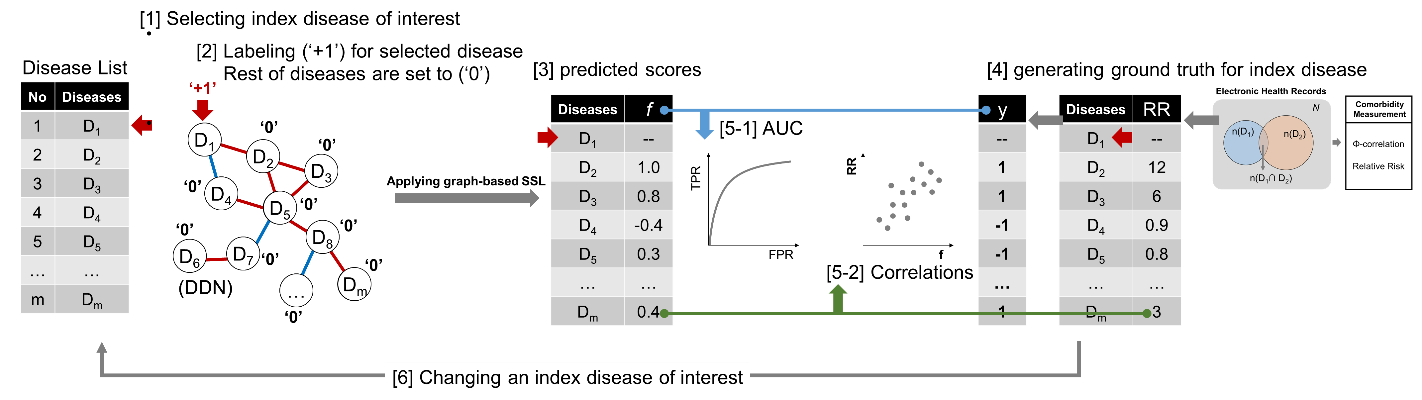
**Supplementary Figure S5.**

**Figure S5. A flow of calculating prediction performances (AUC and rank correlations).** Given the disease network, index disease of interest is selected as query disease. After applying graph-based SSL, we can obtain predicted scores from the network. To calculate AUC and rank correlations, we compared predicted scores and generated ground truths from electronic health records.

**Supplementary Figure S6.**

**A sub-network with directly connected diseases with coronary atherosclerosis
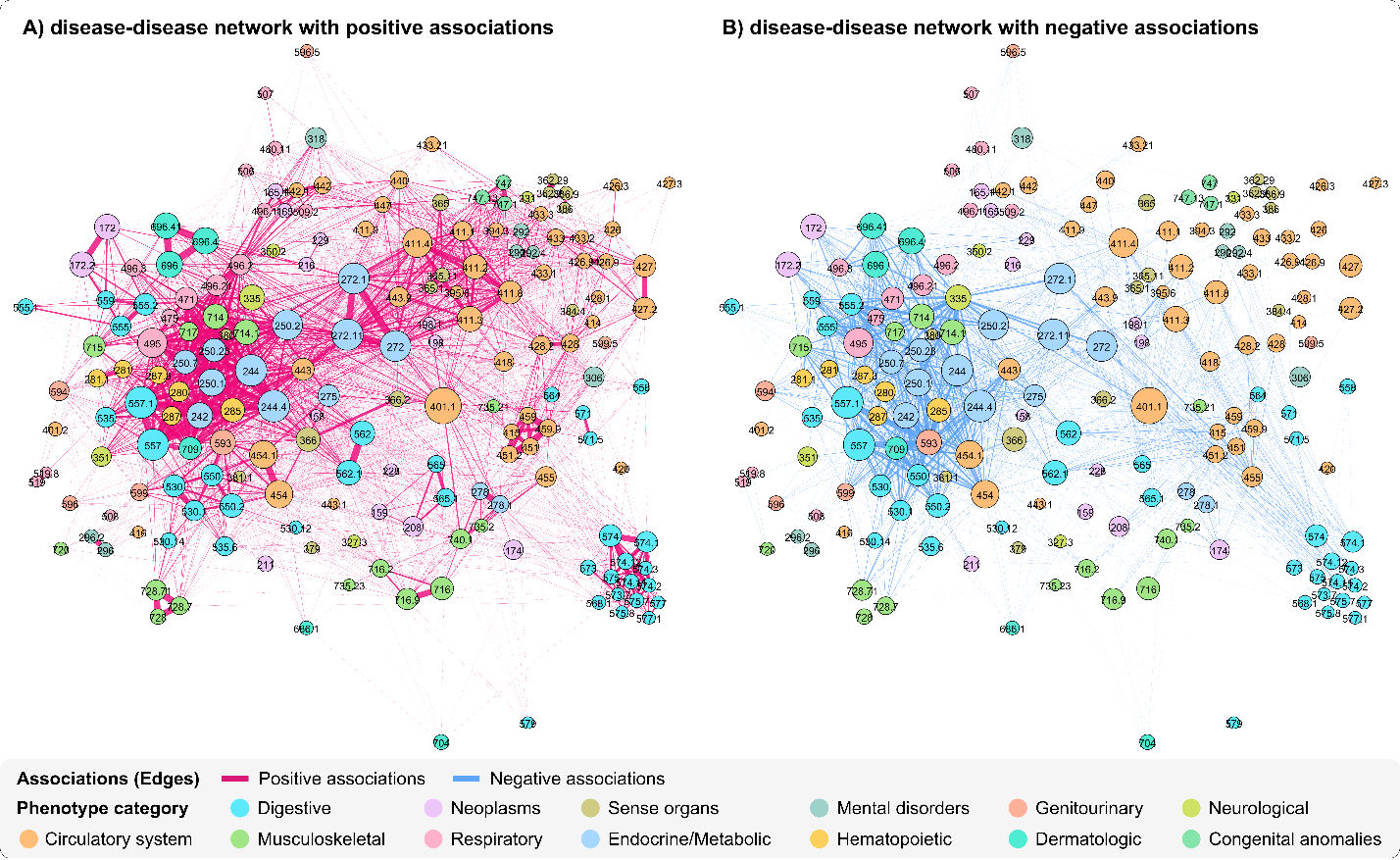
**

**Figure S6. Snapshot of subgraph of signed DDN focused on Coronary atherosclerosis (PheCode 411.4)**
